# Optimising *Chlorella vulgaris* bioflocculation by *Aspergillus Niger* pellets and their application in wastewater treatment and lipid production

**DOI:** 10.1186/s12934-025-02849-z

**Published:** 2025-11-07

**Authors:** Abeer Mohamed, Eman Owis, Gamal Abdel-Fattah, Eladl Eltanahy

**Affiliations:** Botany Department, Faculty of Science, Mansoura University, Mansoura, 35516 Egypt

**Keywords:** Co-culture, Fungal pellet, Bioflocculation, Harvesting, *Aspergillus niger*, *Chlorella vulgaris*, Wastewater treatment

## Abstract

**Background:**

Co-culturing filamentous fungi with microalgae is thought to be a viable method for effective microalgae bioflocculation. Using filamentous fungus makes it possible to produce big, flocculated pellets without adding extra chemicals, which facilitates a variety of uses for the collected biomass. This strategy is popular since it is economical and ecologically beneficial. Most studies examine the two main bio-flocculation strategies to collect microalgae, which are assisted by fungi’s spores or pellets, and their importance in wastewater treatment.

**Results:**

Fungal pellet formation for algal harvesting and wastewater treatment is critically influenced by temperature, glucose concentration, pH, spore suspension concentration, and their interaction, resulting in large pellets (5–28 mm diameter) and achieving 100% algal flocculation efficiency within 18 h under optimized conditions at 26 °C,110 rpm. Depending on the fungal pellets’ biovolume cm^3^/L relative to the algal culture, the algae’s starting density, and the glucose availability. The process demonstrated dual-stage wastewater treatment efficacy: At stage one, biovolume of 107.17 cm³/L achieved 91.88% ammonium and 91.5% phosphate removal (glucose-enhanced phosphate binding), while for blanks 49.1% ammonium, 22.3% phosphate at Stage two (cost-focused), which extends for only 24 h, reduced ammonium by 21.85% and phosphate by 57.18%. In contrast, the blank group only achieved a decrease of 1.5% and 15.9%. However, excessive biovolume counterproductively pollutes water, emphasizing the need for balanced optimization.

**Conclusions:**

Fungal preformed pellets may have a better chance of forming into an efficient bio-flocculation method to harvest microalgae than the fungal spore-assisted approach, which takes a long time, produces a small number of pellets or loose pellets that lack shape or structure, and sometimes growth appears as deposits, also turning algal growth into a yellow color. The pellet-assisted technique shows excellent ability to remove phosphate and ammonium from wastewater. A suitable biovolume must be considered to achieve ideal treatment in wastewater remediation, and adding glucose can improve overall effectiveness.

**Graphical abstract:**

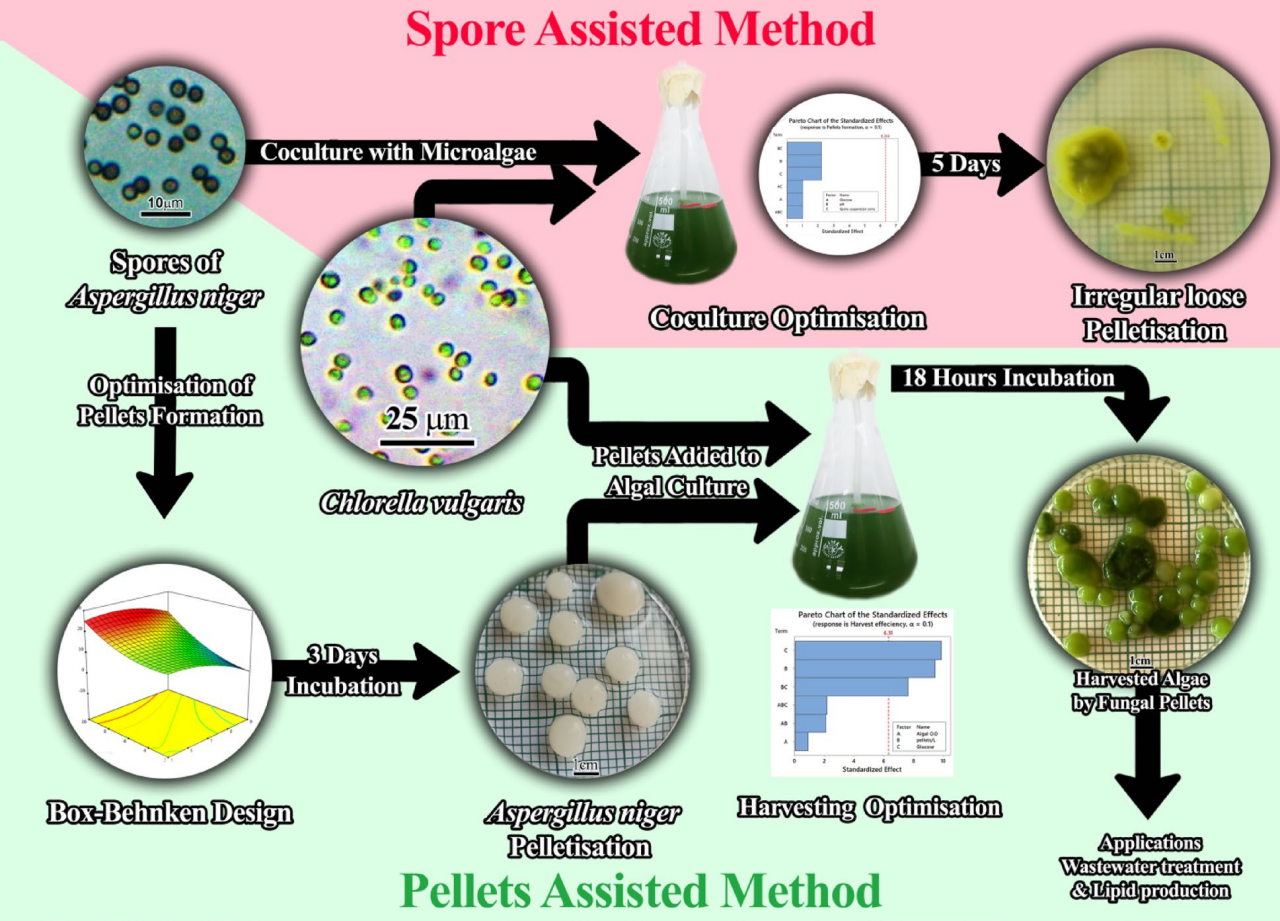

**Supplementary Information:**

The online version contains supplementary material available at 10.1186/s12934-025-02849-z.

## Introduction

 Microalgae are incredibly adaptable organisms that have relatively quick growth, high bioactive compounds (lipid, protein, carbohydrates) contents, through a practical autotrophic ability to grow photosynthetically [1], and use their biomass as feedstock in biofuel [2, 3], pharmaceuticals, feed, and food production [4, 5]. Beyond their biomass value, microalgae also play a critical role in environmental applications, including bioremediation and ecological engineering [6] and wastewater treatment [7]. However, their applications remain constrained by inefficient harvest methods.

One approach in wastewater treatment is the use of high-rate algal ponds (HRAP), a technology that has been investigated for wastewater treatment using microalgae, which can treat sanitary sewage [8]. Moreover, immobilised microorganisms of specific fungi, bacteria, and algae are used as activated sludge due to their high nutrient consumption capacity from waste [9, 10]. So, they are considered a critical breakthrough in removing water pollution and a vital part of the environmental protection ecosystem [11–13].

After treatments, the microalgae cells adsorb (surface binding) or absorb (internal uptake) the pollutants on the cell surface or inside the cell, respectively [14]. However, the pollutants are still in the body of water, and separating the biomass is essential in completing the treatment process. This separation is challenging due to the low cell densities and small cell sizes of microalgal cells, as well as their charged surfaces, which make biomass recovery difficult using traditional methods, including centrifugation, filtration, and flocculation [15, 16]. Centrifugation, while widely used due to its high recovery efficiency, is costly and suitable only for high-value product accumulation, as it requires energy-intensive operations, and it may need a force of 4000 to 14,000 times the gravitational force. In contrast, filtration is species-specific, as it cannot harvest algae species approaching bacterial dimensions like *Chlorella* spp., *Dunaliella* spp., and *Scenedesmus* spp. [17] and prone to membrane fouling, leading to inconsistent performance and higher operational costs, chemical flocculation is also used for harvesting. However, the resulting flocculant-toxins are contaminated, which have environmental risks and cause problems if this biomass is intended for use in food, feed, or pharmaceutical applications. Moreover, flocculants often require secondary treatments, introducing additional costs and complexity to the process [18, 19]. However, it is used as a source of bioproducts and biofuels, as in algal-fungal pellets [20].

These limitations resulted in an increased need for alternative solutions. Recent studies have highlighted the promise of filamentous fungi, especially those integrated as pellets [21], which have been extensively investigated in recent times and indicated much potential impact on flocculation in the future [22, 23]. This new technique involves the co-culturing of algae and filamentous fungi for efficient harvesting by immobilising microalgae through mycelia interactions [24], offering low-cost, high-efficiency, and environmentally friendly harvesting [25]. This technology is considered an innovation in microalgae harvest for high-value products such as proteins [26], carotenoids, carbohydrates, and pigments [27], biofuel production [28, 29], and other bioactive compounds [30]. The resulting large pellets are easy to harvest without additional chemical agents [31, 32]. Filamentous fungi like *Aspergillus* sp., *Mucor* sp., and *Penicillium* sp. have self-pelletisation capabilities and are highly effective in harvesting microalgae [19, 32, 33]. Fungal-algal pellets can be formed using two methods: the fungal spore-assisted method and the fungal pellet-assisted method [34].

There are many factors affecting pellet formation. Firstly, pH value, which is an essential factor that has different effects according to the strain and genotypes of cells used [35, 36]. Some species favour an alkaline pH to form pellets, like *Penicillium chrysogenum*, which forms pellets at pH >7.4. In contrast, other fungi, such as *Rhizopus oryzae*, form pellets only at acidic conditions (pH 3–7) [20, 21, 37, 38]. As a result, selecting specific fungal species that can adapt to a wide range of pH is necessary because microalgae require an alkaline environment to grow well [39]. On the other hand, some reports mentioned that pH slightly affected flocculation [40–42]. Secondly, the initial spore suspension concentration is the same as the pH. The ideal initial concentration depends on the strain and also affects the growth and morphology of the pellets [35], as at a certain level of spore concentration, the formation of dispersed mycelia occurs, but they cannot form the pellets [43, 44]. There is a significant inverse relationship between initial spore concentration and pellet size. This correlation could help to understand the characteristics of mycelial pellets and help produce pellets [45]. Also, temperature directly affects the formation and morphology of the pellets [35]. In general, the increase in medium temperature leads to sufficient kinetic energy to accelerate the flocculation process [46]. On the other hand, in some species, such as *Penicillium* sp., the fungal spores obtain higher metabolic activity when the temperature is above 33 °C. Still, the optimum cultivation temperature for most microalgae is between 20 and 30 °C [47]. Also, high temperatures can deform the algal cell wall and break the structure of the cell distribution, which is confirmed by using a scanning electron microscope (SEM) analysis. Finally, glucose is a suitable organic carbon source for fungi and algae to be utilised [12], which impacts fungal growth and pelletisation. Higher initial glucose concentration is found to shorten the pelletisation process’s cultivation period, resulting in much lower pH [20]. This study aims to optimize fungal pellet production and apply it to microalgal harvesting in wastewater treatment. The optimized process resulted in 100% algal recovery and over 90% nutrient removal, significantly outperforming traditional methods in both efficiency and environmental sustainability. So it supports the continued development of fungal-algal co-cultivation as a scalable, low-cost, and eco-friendly solution for microalgal biomass recovery.

## Materials and methods

### Sample collection

Municipal wastewater samples were collected from the wastewater treatment unit at Dakahlia Electricity Company, Talkha, Dakahlia Governorate, Egypt, 31 °03’ 45.8 N 31 °23’ 45.1” E in sterilized bottles, then transferred immediately to the laboratory.

### Isolation and identification of fungal strains

The collected samples were streaked on Potato Dextrose Agar (PDA) plates as the growth medium for fungi [48]. The isolated strains were then assessed for their capability to grow on the standard algal BBM medium [49] supplemented with glucose. The morphological characteristics of the selected candidate’s mycelia were examined using an optical microscope identified as *A. niger* strain, then for DNA molecular identification, the ZYMORESEARCH™ kit (Quick-DNA™ Fungal/Bacterial Miniprep Kit) was used, Where COSMO PCR RED Master Mix protocol involves preparing a master mix with COSMO PCR RED Master Mix, primers, nuclease-free water, and DNA template, followed by PCR amplification with an initial denaturation at 95 °C for 2 min, 25–35 cycles of denaturation (95 °C, 15 s), annealing (primer Tm − 5 °C, 20 s), and extension (72 °C, 30–60 s), and a final extension at 72 °C for 1 min. The PCR products are then analyzed by agarose gel electrophoresis.

### Isolation and identification of algal strains

The previously collected samples were utilized to isolate algal strains in the laboratory by spreading the sample onto the original Bold’s Basal Medium (BBM) as outlined by Bischoff H [49]. The unialgal cultures were subsequently maintained in a BBM broth medium after morphological identification. Furthermore, the genomic DNA from algal strains was isolated and purified using the DNeasy ^@^Plant Kit (Germany) following the manufacturer’s protocols. DNA concentration was measured using a spectrophotometer at 260 nm and 280 nm, and quality was confirmed by running 5 µL on a 1% agarose gel with ethidium bromide staining. The 18 S rDNA gene was amplified using specific primers (DSs: 5′-GCAGGAGAGCTAATAGGA-3′ and DPs: 5′-GTAGAGGGTAGGAGAAGT-3′) in a 25 µL PCR reaction containing 1 µL DNA (10 ng), 0.2 µL Taq DNA polymerase, 5 µL 10x Taq buffer, 2.5 µL dNTPs, 2.5 µL of each primer, and nuclease-free water. PCR conditions included initial denaturation at 94 °C for 4 min, 35 cycles of denaturation (94 °C, 50 s), annealing (58 °C, 60 s), and extension (72 °C, 1 min), followed by a final extension at 72 °C for 7 min. Amplified DNA bands were excised and purified using a DNA gel extraction kit (Sigma-Aldrich, Germany).

### Fungal pelletisation

#### Preparation of fungal spore suspension

*A. niger* was kept on a potato dextrose agar slant [50, 51]. The slant was transferred to a Petri dish and incubated for 5 days, then washed with distilled water to extract the spore suspension, and the number of spores in the suspension was counted with an optical microscope using a hemocytometer (0.1 mm deep) slide.

#### Factors affecting the formation of fungal pellets

*A. niger* was evaluated for its ability to form pellets on the BBM medium supplemented with glucose. Several factors were observed using one factor at a time to influence pellet formation, including initial inoculum concentration, pH, glucose supplements, and temperature [20, 52, 53]. All treatments proceeded in triplicate using sterilised 250 mL conical flasks containing 100 mL of BBM medium, incubated on a horizontal shaker at 110 rpm. The first set included different counts of fresh fungal spores 1 × 10^5^, 1 × 10^6^, 1 × 10^7^, 1 × 10^8^, and 1 × 10^9^ spores/L, while the second set contained glucose concentrations of 2, 5, and 10 g/L. Furthermore, the third set contained different initial pH values (4, 5, and 6), and the fourth set was incubated at temperatures of 26, 30, and 35 °C. On the other hand, the control treatment conditions were glucose 10 g/L, spore suspension concentration 1 × 10^5^, pH = 6, and temperature 26 °C. The results were analysed statistically using the SPSS 16 programme (©2007 SPSS Inc., Chicago, IL, USA). The one-way ANOVA was performed, followed by Duncan multiple range tests to determine significant differences between means.

#### Physicochemical parameters optimisation using Box-Behnken design

A statistical optimisation design, the Box-Behnken design, was employed to determine the best cultivation conditions for interacting physicochemical factors during fungal pellet production. The Box-Behnken design matrix, as shown in Table 1, consisted of three levels for each variable, resulting in 27 trials for four factors with three tested levels for each parameter. The trials were conducted in the same incubation conditions previously mentioned, but with slight modifications by following the matrix design for the studied factors.

**Table 1:** Variables and their levels used to design Box-Behnken experiments for spore suspension concentration, glucose concentration, pH, and temperature

Levels			
Spore concentration (spores/L)	1 × 10^5^	1 × 10^7^	1 × 10^9^
Glucose concentration (g/L)	2	6	10
pH	4	5	6
Temperature (°C)	26	30. 5	35

### Fungi-algae co-culture

#### Spore-assisted method

The critical factors (initial spore suspension concentration, glucose concentration, and pH) that can affect the formation of algal-fungal pellets and harvest efficiency were studied and optimised using a 2-level full factorial design, which formed eight different trials with three different parameters, where each parameter was investigated in low (−1) and high level (+ 1) as shown in Table 2. All experiments were conducted for 5 days in 250 mL Erlenmeyer flasks placed on an orbital shaker at 110 rpm at a temperature of 26 ˚C by adding one ml of fungal spore suspension, and 10 mL enriched seed cultures were inoculated on 100 mL BBM medium [34].

**Table 2:** Variables and their levels used for the co-culture experiment using a 2-level full factorial design

Variables	Mini-level (−)	Maxi-level (+)
Glucose (g/L)	2	10
pH	4	6
Spore suspension (spores/L)	1 × 10^5^	1 × 10^9^

#### Pellets-assisted method

This method for testing the performance of fungal pellets to harvest algal cells under specific factors, including algal culture concentration, fungal pellets biovolume cm^3^/L algal culture, where pellets’ biovolume is calculated as the volume of a sphere 4/3 π r^3^ due to their near-spherical morphology., and glucose concentration. The conditions for harvesting of algal cells using fungal preformed pellets were optimised using a 2-level full factorial design, which was applied to enhance the harvest ability where the *A. niger* pellets were incubated with *C. vulgaris.* The design produced eight trials with three different parameters, and each parameter was investigated at low (− 1) and high levels (+ 1), as shown in Table 3. These treatments were incubated at a temperature of 26 ˚C, in 250 mL Erlenmeyer flasks placed on an orbital shaker at 110 rpm.

**Table 3:** Variables and their levels used for the co-culture experiment using a 2-level full factorial design

Variables	Mini. level (−)	Maxi. level (+)
Glucose concentration (g/L)	2	10
Fungal pellets biovolume cm^3^ /L of algae	107.17	321.5
Algal culture concentration (OD)	1	2

### Harvest efficiency

Harvest efficiency was calculated based on the cell counts, which were determined by directly counting the cells using a light microscope at 400X magnification with a 0.1 mm deep hemocytometer. Optical density was also measured using a spectrophotometer [54] before and after fungal-algal pellet formation to evaluate harvesting efficiency. Harvest efficiency was calculated by counting algal cells before adding fungal pellets to the algal culture and at the end of the experiment using the following formula.

$$\begin{array}{l}{\rm{Harvest efficiency \% = (100}}\\ - {\rm{(final algal count after 18h/initial algal count))}}\end{array}.$$

### Wastewater treatment

The effectiveness of algal growth in the wastewater as a medium was assessed. To design the medium, some physical and chemical properties were determined, such as pH, TDS, conductivity, and salinity, as well as element concentrations such as phosphate and ammonium. Furthermore, some essential trace elements were measured using an inductivity-coupled plasma (iCAP™ 7000 Plus Series ICP-OES, Thermo Scientific™). Waste analysis was conducted [55]. The enriched seed cultures were then inoculated at a 10% volume of inoculum to the volume of medium in 100 mL wastewater liquid medium placed in 250 mL Erlenmeyer flasks. The flasks were set up for bubbling under continuous illumination of cool-white fluorescent light at an intensity of 3600 lx. And at a temperature of 26 ± 2 °C.

### Analysis of wastewater nutrients

The primary objective was to assess the efficiency of nutrient removal, specifically targeting ammonium (NH₄⁺-N) and total phosphorus (TP), which were analyzed before and after treatment using the Hach DR 2010 Spectrophotometer. Ammonium levels were measured using Nessler’s reagent [56]. Phosphate concentrations were analyzed via the direct stannous chloride method using standard methods described by the American Public Health Association [57, 58]. All experiments were performed in triplicate to ensure reliability, and the average values were reported. The removal efficiency percentage was calculated by using the following equation:

$$\begin{array}{l}{\rm{Removal efficiency percentage }}\% \\\quad = {\rm{ }}\left( {\left( {{\rm{I}} - {\rm{F }}/{\rm{F}}} \right) \times {\rm{1}}00\% } \right).\end{array}.$$

Where I = nutrients’ initial concentration at the beginning of the experiment; F nutrients’ final concentration after treatment.

For testing the ability of the symbiotic system of *C. vulgaris* and *A. niger* for wastewater treatment. This technique was performed in two stages. In the first stage, *C. vulgaris* was cultivated in wastewater under lighting conditions of 3,600 lx for 6 days. It was used as a growth medium, effectively utilizing the nutrients present in the wastewater, thereby treating the water. Meanwhile, *A. niger* pellets were used to harvest the algal cells for 24 h, with experiments conducted in 250-mL Erlenmeyer flasks on a shaker at 110 rpm, maintaining the same temperature 26 ± 2 °C and this resulting in the formation of fungi-algae pellets and treated supernatant., moreover fungal pellets biovolume/L algal cultured on wastewater and glucose supplements were observed using one factor at a time to influence treatment process at conditions mentioned earlier.

In the second stage, these pellets were filtered and tested for their potential reuse as immobilized cells to reduce costs by adding them to fresh wastewater samples for 24 h under the same conditions as the first stage. Their biovolume was the fungal pellets’ biovolume/L of algal culture on wastewater, regarded as a key factor that could influence their effectiveness. Throughout the experiment, all flasks were conducted in triplicate, along with a control that contained only normal microflora and had no treatments. All flasks were carried out under the same conditions to measure phosphate and ammonium levels.

### Lipid extraction

The harvested biomass was centrifuged at 4000 rpm for 10 min, and then the collected pellets were lyophilized. The lipid is extracted by using the chloroform and methanol (2:1) method [59]. The weight of lipids was expressed in mg/L and dry cell weight% (DCW%), which is calculated using the following equation.

$${\text{Lipid content of }}\left( {\% {\text{ DCW}}} \right)=\left( {{\text{LD}}/{\text{ LB}}} \right) \times 100$$

Where LD: the weight of dried lipid after solvent evaporation, LB: Initial lyophilized biomass for lipid extraction, and % of DCW: % of dry cell weight [60].

### Statistical analysis

Data were investigated using SPSS software and were characterized by the mean ± standard deviation (SD) of the results of triplicate experiments. One-way ANOVA was applied to analyse the significant differences between factors using Duncan’s test at a significant level of *p* ≤ 0.05. Factor optimisation was performed through Box-Behnken at a significant level of *p* ≤ 0.05, which consisted of variables and responses that were evaluated statistically and subjected to evaluate the effectiveness of the model by Design Expert V.13, which was used to analyze the variation (ANOVA) of the produced models [34]. Also a 2-level full factorial method using Minitab^©^ software at a 90% confidence level. To moderate the error risk. This adjustment aligns with similar methodological approaches in the field to balance accuracy with the practical need, and this agrees with [61] whose study employed a 90% confidence level using a 2-level full factorial design to evaluate the significance of the main effects and interactions among the bioremediation strategies, also [62] used 90% confidence to analyze the significance at the Plackett–Burman design.

## Results

### Isolation and identification of the fungal strain

**Fig. 1:**
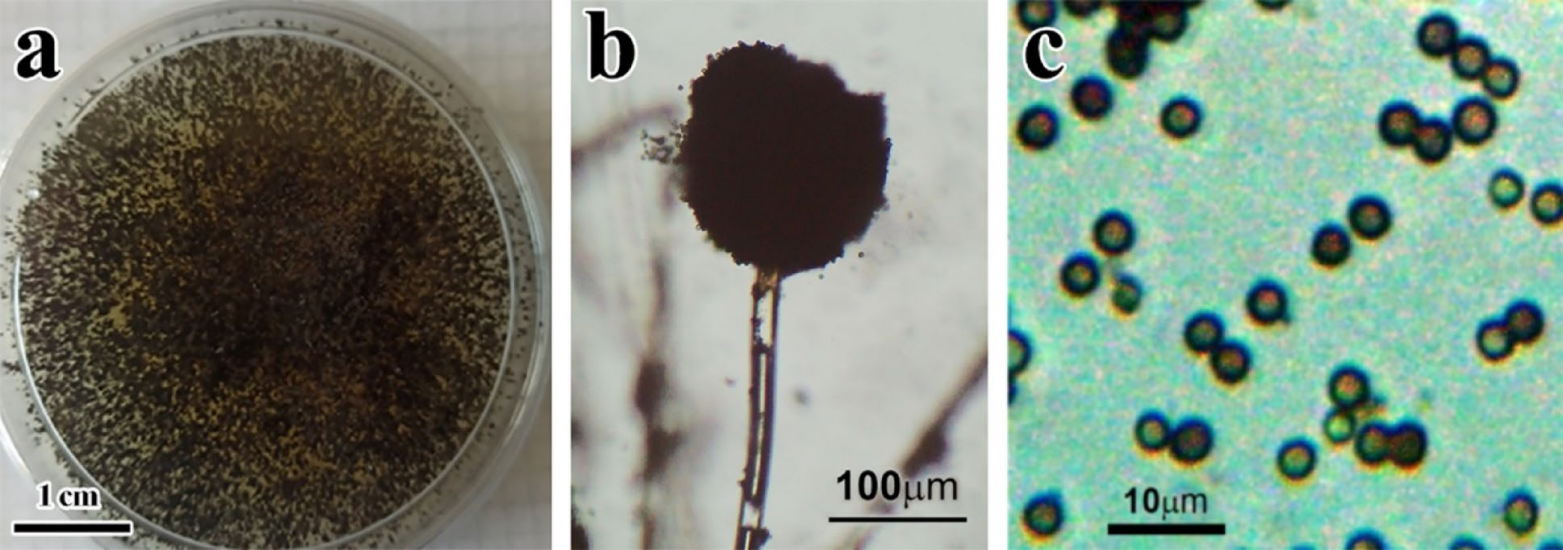
**a** Morphological identification of *A. niger* **a** colony morphology on potato dextrose agar medium (PDA) after 5–7 days as it appears in black conidial spores (scale bar 1 cm), **b** light microscobe of conidiophore structure and conidium (scale bar 100 μm), and **c** High magnification view of thick-walled *A. niger* black spores (scale bar 10 μm)

**Fig. 2:**
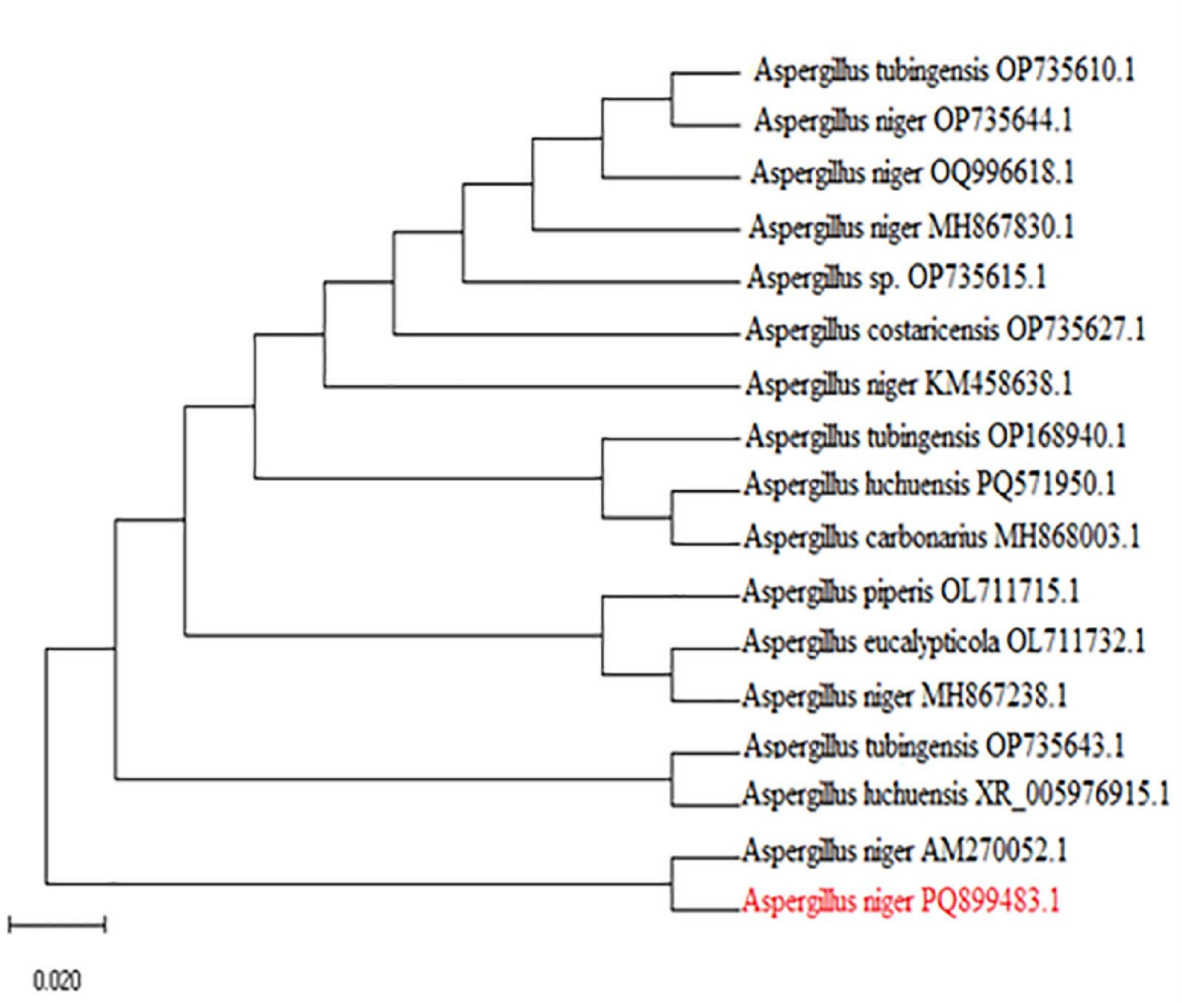
Phylogenetic tree for an unknown fungal species, which confirmed that the fungus species is *Aspergillus niger*

In early experiments, the selected isolated filamentous fungus was identified as *A. niger* by using morphological characters [63, 64]. The growth patterns, as it initially appears white, mycelium changes into black colour after a few days due to producing conidial spores (Fig. 1a). Furthermore, microscopic examination shows a dark brown, globose conidial head that produces dark brown to black spores. Hyaline or turning dark towards the vesicle. As shown in Fig. 1b, and c. The fungus identification was confirmed using molecular identification, shown in Fig. 2 (with accession number PQ899483).

### Isolation and identification of the algal strain

**Fig. 3:**
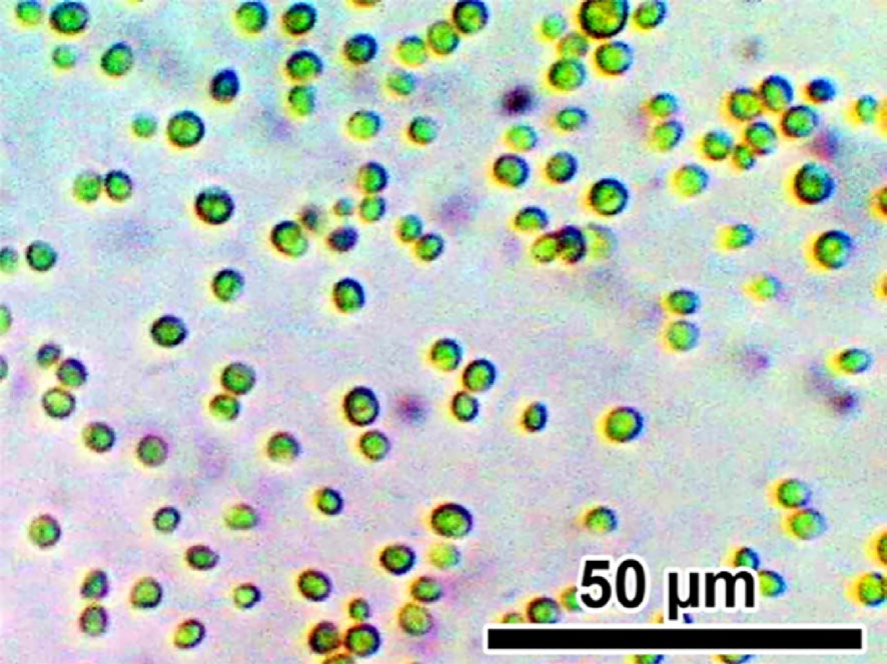
Green, unicellular, non-motile, and has a spherical shape without flagella, algal strain, which is identified as *C. vulgaris* (scale bar 50 μm)

**Fig. 4:**
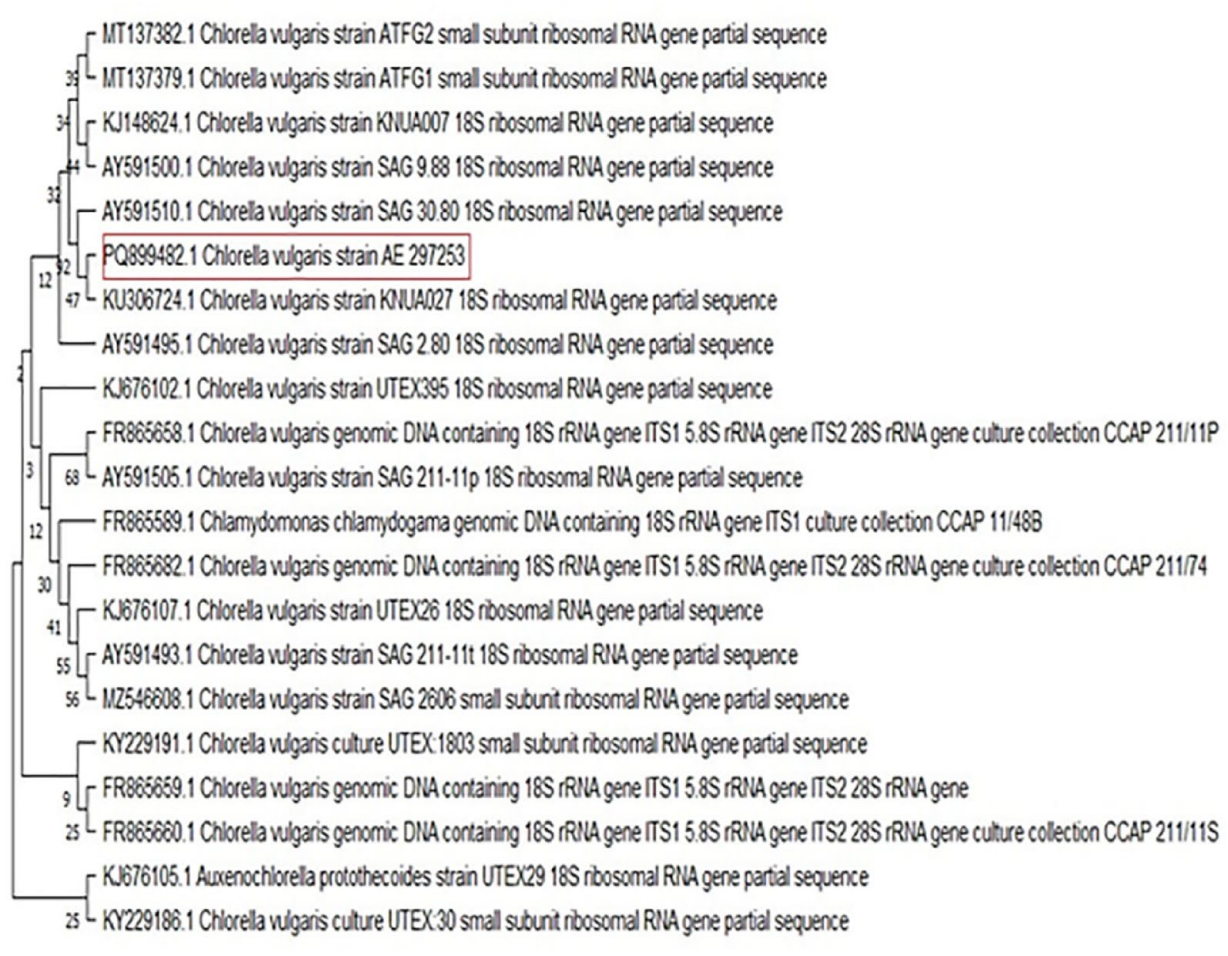
Phylogenetic tree for an unknown algal species, which is identified as *C. vulgaris*

According to algae morphological characteristics, the pure microalga strain was preliminarily identified as *C. vulgaris*, as it is green, unicellular, non-motile, and has a spherical shape with a diameter size ranging from 2.6 to 4.3 μm, parietal and cup-shaped chloroplast without flagella (Fig. 3). This is confirmed by molecular identification (Fig. 4) with accession number PQ899482 and the pure isolate submitted in Mansoura University Algal Collection with code MU-BB-59.

### Fungal pelletisation

#### Factors affecting the formation of fungal pellets

*A.niger* showed a remarkable ability to self-pelletise in a standard algal BBM medium supplemented with glucose after three days under laboratory conditions. Initial spore suspension concentration, pH, supplemented glucose concentration, and temperature significantly affected pellet formation, varying pellet counts and sizes.

**Fig. 5:**
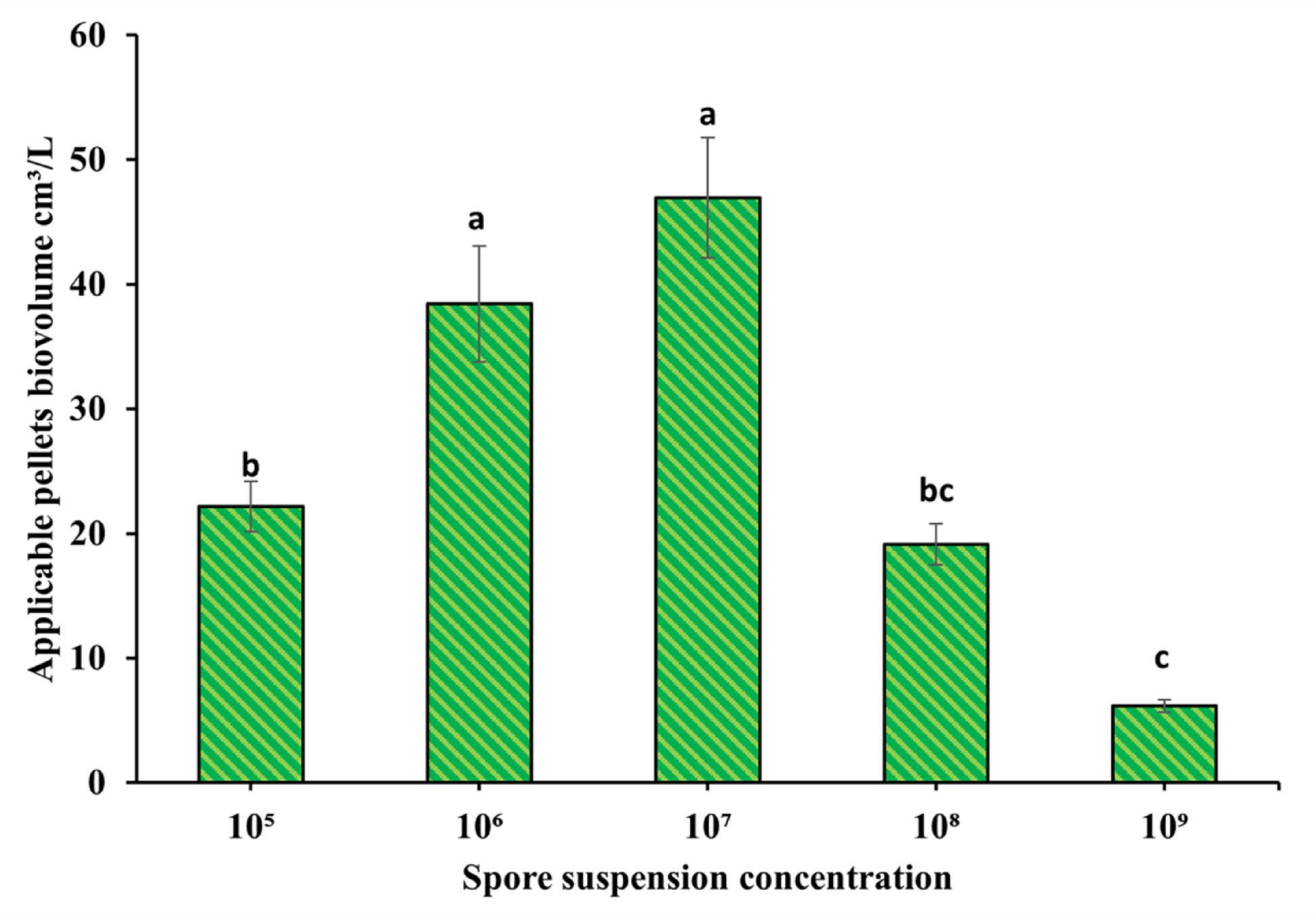
Spore suspension concentration effect on *A. niger* pellets volume formation

The effect of initial spore inocula on fungal pellet formation was studied in the treatments, and the formation of pellets was not a perfect structure and rarely appeared as a fluffy mycelium or clumps, which were not considered in the counting and the calculations of pellet biovolume, which was expressed as the applicable pellet biovolume. As shown in Fig. 5 The inoculum concentration ranged from 1 × 10^5^ to 1 × 10^9^ spores/L, and the pellet formation took three days to appear. The fungal compact regular pellets were formed with a significant increase in the biovolume, reaching up to 47 ± 4.8 cm^3^/L when the concentration of the inoculum was 1 × 10^7^ spores/L. In contrast, inoculum concentration 1 × 10^6^ spores/L gave the second-best-used pellets biovolume results of 38.4 ± 4.6 cm^3^/L with a moderate pellets biovolume; both 1 × 10^6^ and 1 × 10^7^ have the same best significance. In contrast, the biovolume was significantly reduced to its minimum value of 6.16 ± 0.5 cm^3^/L only when the inoculum concentration was 1 × 10^9^ spores/L. Also, the pellets’ biovolume reached 22.1 ± 2 and 19.15 ± 1.66 cm^3^/L for 1 × 10^5^ and 1 × 10^8^ spores/L, respectively.

**Fig. 6:**
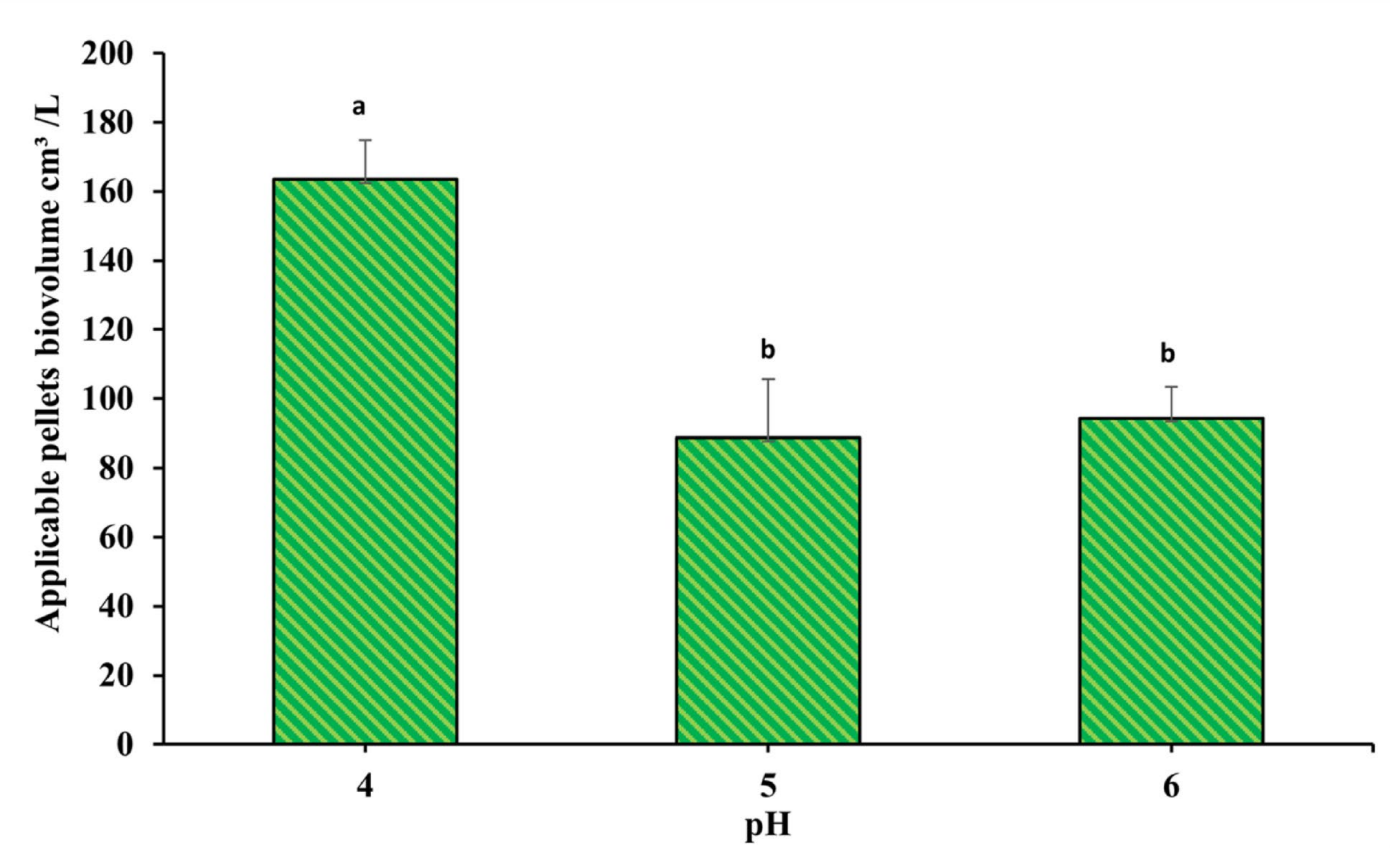
pH effect on *A. niger* pellets volume formation

The pH factor showed a significant difference in pellet volume formation. The pH = 4 showed the highest biovolume of 163.45 ± 11.34 cm^3^/L with a significant increase compared to the other treatments, while the pellets’ biomass mean volume showed a significant decrease in both pH = 5 and 6, resulting in 88.66 ± 17 and 94.3 ± 8.975 cm^3^/L, respectively, as shown in Fig. 6.

**Fig. 7:**
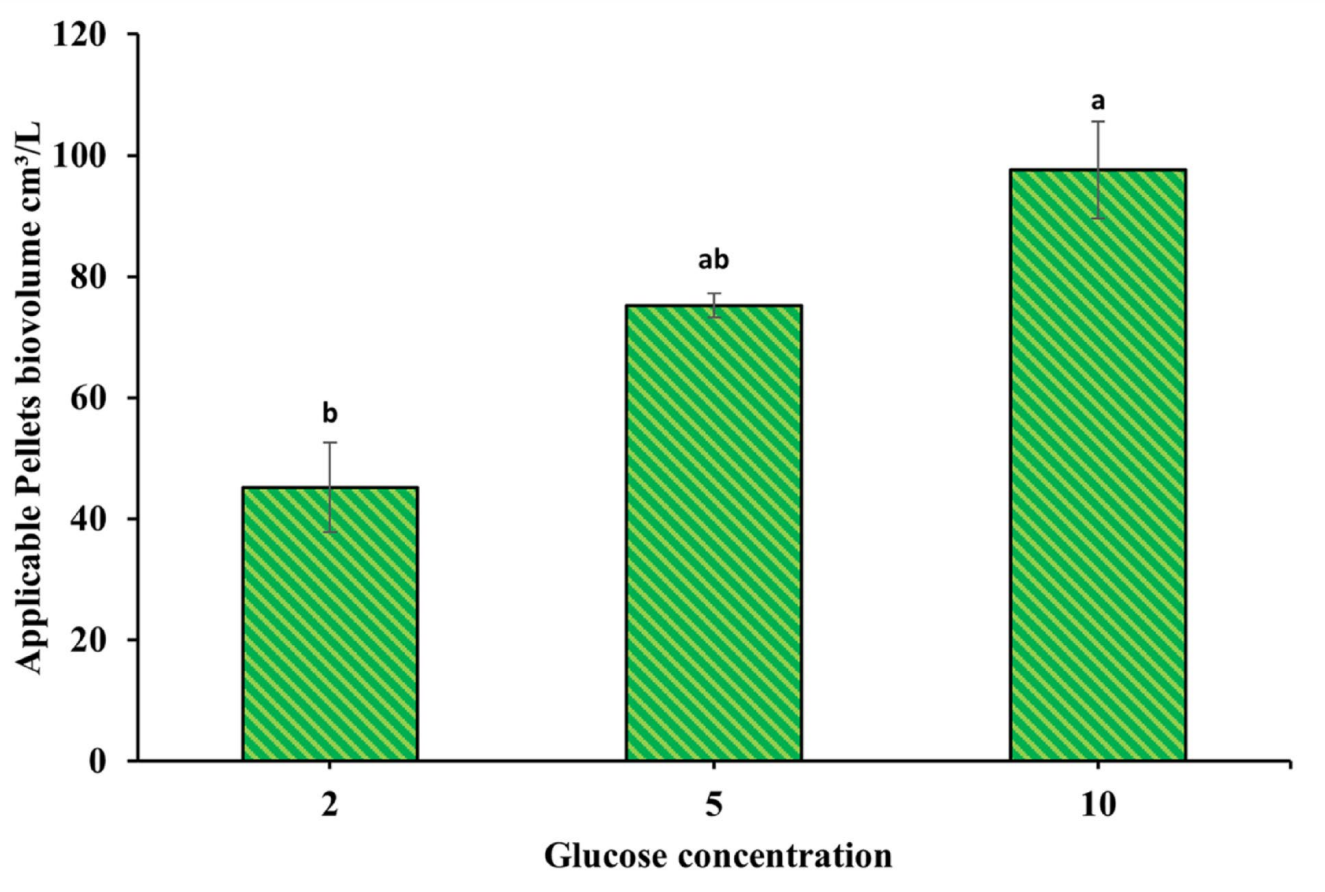
Supplemented glucose effect on *A. niger* pellets volume formation

Glucose was observed to be a vital factor for fungal growth, and pelletisation forms strictly at a concentration equal to 10 g/L. It gave intact large pellets in a few pellets with high biovolume equal to 97.6 ± 8 cm^3^/L, while glucose concentrations of 5 g/L and 2 g/L resulted in lower biovolume, which was 75.1 ± 1.99 and 45.2 ± 7.38 cm^3^/L, respectively (Fig. 7).

**Fig. 8:**
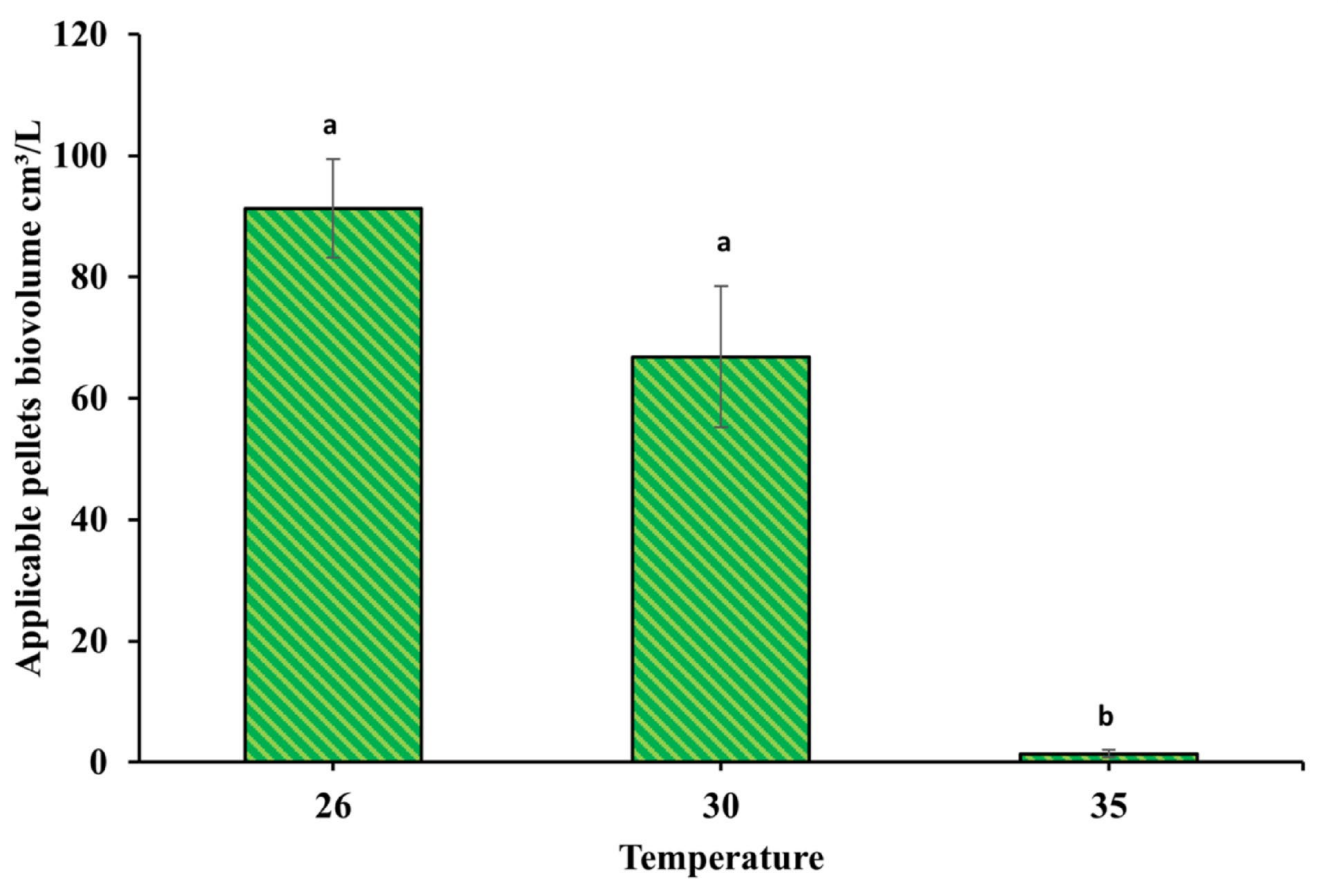
Temperature effect on *A. niger* pellets volume formation

The studied temperature degrees showed that 26 and 30 ˚C have the same significance, as 26 ˚C yielded a higher biovolume of 91.29 ± 8.1 cm³/L, whereas at 30 ˚C it yielded 66.85 ± 11.6 cm³/L. On the other hand, a dramatic effect happened when the temperature was raised to 35 ˚C. It negatively affected pellet density and biovolume as it formed large pellets with loose structures, and at the same time, a few applicable pellets with a mean volume of 1.4 ± 0.5 cm^3^/L were formed (Fig. 8).

#### Optimisation of parameters on A. niger pellet formation

Based on previous single variable experiments, the effects and interaction of pH, temperature, glucose concentration, and initial spore suspension on the formation of *A. niger* pellets using the Box-Behnken design (BBD) which considered as popular Response Surface Methodology (RSM) design and are tabulated in Table 4, which includes the target response due to the changes in the physicochemical factors.

**Table 4:** Box–Behnken design to study the effects of initial spore suspension concentration, glucose, pH, temperature, and the interaction between them

Run no.	Different variables				Response
	Temperature (ºC)	pH	Spore sus. (spores/L)	Glucose (g/L)	Pellets biovolume (cm^3^/L)
1	30. 5	6	1 × 10^7^	2	10.19
2	30.5	5	1 × 10^7^	6	3.55
3	35	4	1 × 10^7^	6	7.17
4	30.5	6	1 × 10^7^	10	10.72
5	35	6	1 × 10^7^	6	0
6	30.5	6	1 × 10 ^9^	6	0
7	30.5	4	1 × 10^7^	2	21.87
8	**26**	**5**	**1 × 10** ^**7**^	**10**	**40.18**
9	30.5	6	1 × 10^5^	6	0
10	30.5	4	1 × 10 ^9^	6	0
11	26	5	1 × 10^7^	2	8.37
12	30.5	5	1 × 10^7^	6	4.79
13	30.5	5	1 × 10^5^	6	21.33
14	30.5	5	1 × 10^5^	10	0
15	35	5	1 × 10 ^9^	6	0
16	26	6	1 × 10^7^	6	18.54
17	30.5	5	1 × 10 ^9^	10	0
18	30.5	5	1 × 10 ^9^	2	0
19	30.5	5	1 × 10^7^	6	1.22
20	26	5	1 × 10 ^9^	6	0
21	35	5	1 × 10^7^	2	0
22	26	5	1 × 10^5^	6	0
23	35	5	1 × 10^7^	10	0
24	35	5	1 × 10^5^	6	0
25	30.5	5	1 × 10^5^	2	12.87
26	26	4	1 × 10^7^	6	23.99
27	30.5	4	1 × 10^7^	10	33.24

The experiment showed that for the maximum pellets biovolume, which produced pellets with a biovolume of 40.18 cm^3^/L in trial number 8, with a temperature of 26 °C, pH of 5, spore suspension concentration of 1 × 10^7^ spores/L, and glucose concentration of 10 g/L. The second-best run was number 27 at a temperature of 30.5 °C, pH of 4, spore suspension concentration of 1 × 10^7^ spores/L, and glucose concentration of 10 g/L, which produced a pellet biovolume of 33.24 cm^3^/L Then, run number 26 is the third-best condition for pellet biovolume, equal to 23.9 cm^3^/L. On the other hand, 13 trials did not form any applicable pellets.

The main effects, low-order interactions, and higher-order interactions were dominated by a multivariable system [65] and the effectiveness of the model is tabulated as follows Table 5.

**Table 5:** ANOVA analysis of the model (asterisk for significance)

Source	Sum of squares	df	Mean square	F-value	*p*-value
Model	2654.18	14	189.58	3.25	0.0238*
A-Temperature	586.87	1	586.87	10.06	0.0080*
B-pH	387.25	1	387.25	6.64	0.0243*
C-Spore suspension	97.49	1	97.49	1.67	0.2205
D-Glucose	79.28	1	79.28	1.36	0.2664
AB	0.7357	1	0.7357	0.0126	0.9124
AC	0.0000	1	0.0000	0.0000	1.0000
AD	252.88	1	252.88	4.33	0.0594
BC	113.78	1	113.78	1.95	0.1879
BD	29.40	1	29.40	0.5039	0.4913
CD	41.42	1	41.42	0.7099	0.4160
A²	21.32	1	21.32	0.3654	0.5568
B²	349.85	1	349.85	6.00	0.0307*
C²	192.54	1	192.54	3.30	0.0943
D²	253.62	1	253.62	4.35	0.0591
Residual	700.12	12	58.34		
Lack of fit	693.54	10	69.35	21.08	0.0461*
Pure error	6.58	2	3.29		
Cor total	3354.30	26			

The calculated responses could be mathematically modelled as follows:

$$\begin{gathered} {\mathbf{Pellets}}{\text{ }}{\mathbf{biovolume}}={\text{316}} - {\text{4}}.{\text{4 temp}} - {\text{98}}.{\text{4 pH}} \hfill \\ \quad +{\text{3}}.{\text{9 spores}}+{\text{9}}.{\text{52 glucose}}+0.0{\text{99 temp}}*{\text{temp}} \hfill \\ \quad +{\text{8}}.{\text{1}}0{\text{ pH}}*{\text{pH}} - {\text{1}}.{\text{5}}0{\text{2 spores}}*{\text{spores}} \hfill \\ \quad +0.{\text{431 glucose}}*{\text{glucose}} - 0.0{\text{95 temp*pH}} \hfill \\ \quad +0.000{\text{ temp}}*{\text{spores}} - 0.{\text{442 temp}}*{\text{glucose}} \hfill \\ \quad +{\text{2}}.{\text{67 pH}}*{\text{spores}} - 0.{\text{678 pH}}*{\text{glucose}} \hfill \\ \quad +0.{\text{4}}0{\text{2 spores}}*{\text{glucose}}. \hfill \\ \end{gathered} $$

The *p-*value of 0.0238 indicates the significance of the model at a confidence level of 95%. The R² value of 79.13% indicates that the model explains 79.13% of the variability in the data and provides a good fit for the observed results.

In general, a *p*-value < 0.05 indicates the significance of a variable. This means that the temperature (*p* = 0.0080) and pH (*p* = 0.0243) significantly affected the response. Also, the *p*-value of the pH quadratic effect is 0.0307 (Table 5), indicating that the pH quadratic effect had a great impact on the response value of all items, and the model design was confirmed by the model’s F value of 3.25 with a significant lack of fit equal to 0.0461.

**Fig. 9:**
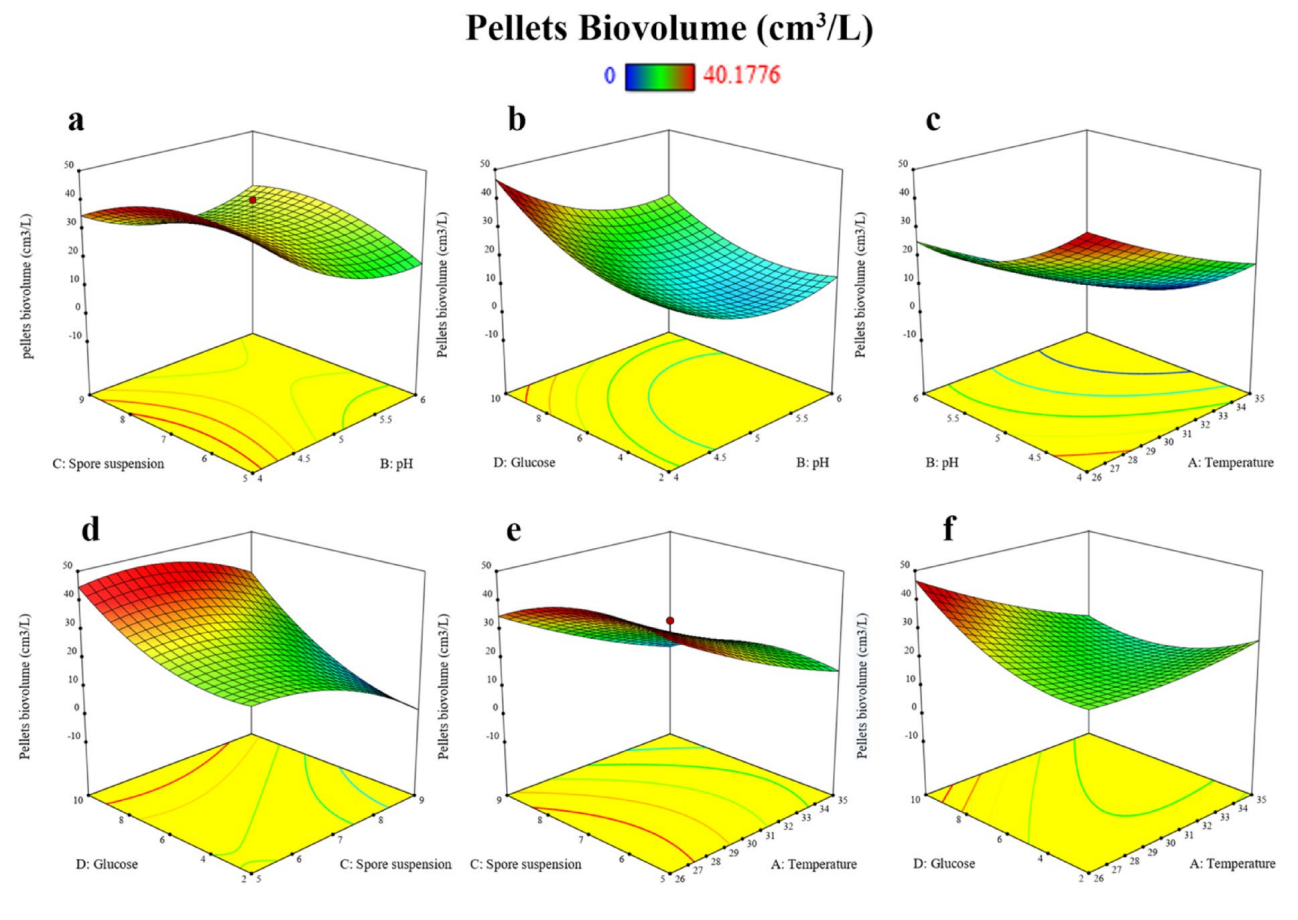
Three-dimensional response surface plot for the interactive process variables on pellet biovolume **a** pH and spore suspension, **b** pH and glucose concentration, **c** Temperature and pH, **d** Sporesuspension and glucose concentration, **e** Temperature and sporesuspension, **f** Temperature and glucose concentration

Three-dimensional response surface plot for the interaction between pH, temperature, glucose, and spore suspension showed that the interaction between pH and spore suspension was found to be effective on pellets’ biovolume (Fig. 9a) with higher biovolume at spore concentration ranges from 1 × 10^5^ to 1 × 10^8^ spores/L and pH value between 4 and 4.5. While the interaction between pH and glucose concentration (Fig. 9b) showed that the biovolume is reciprocally correlated with the pH value, and the optimal glucose concentration is 10 g/L. The optimal pH range in this case is between 4 and 4.5. In the interaction between pH and temperature, the optimal temperature range was 26–28 °C (Fig. 9c). In the other RSM figures Fig. 9d, e, and f, the trend of the interactions was the same.

**Fig. 10:**
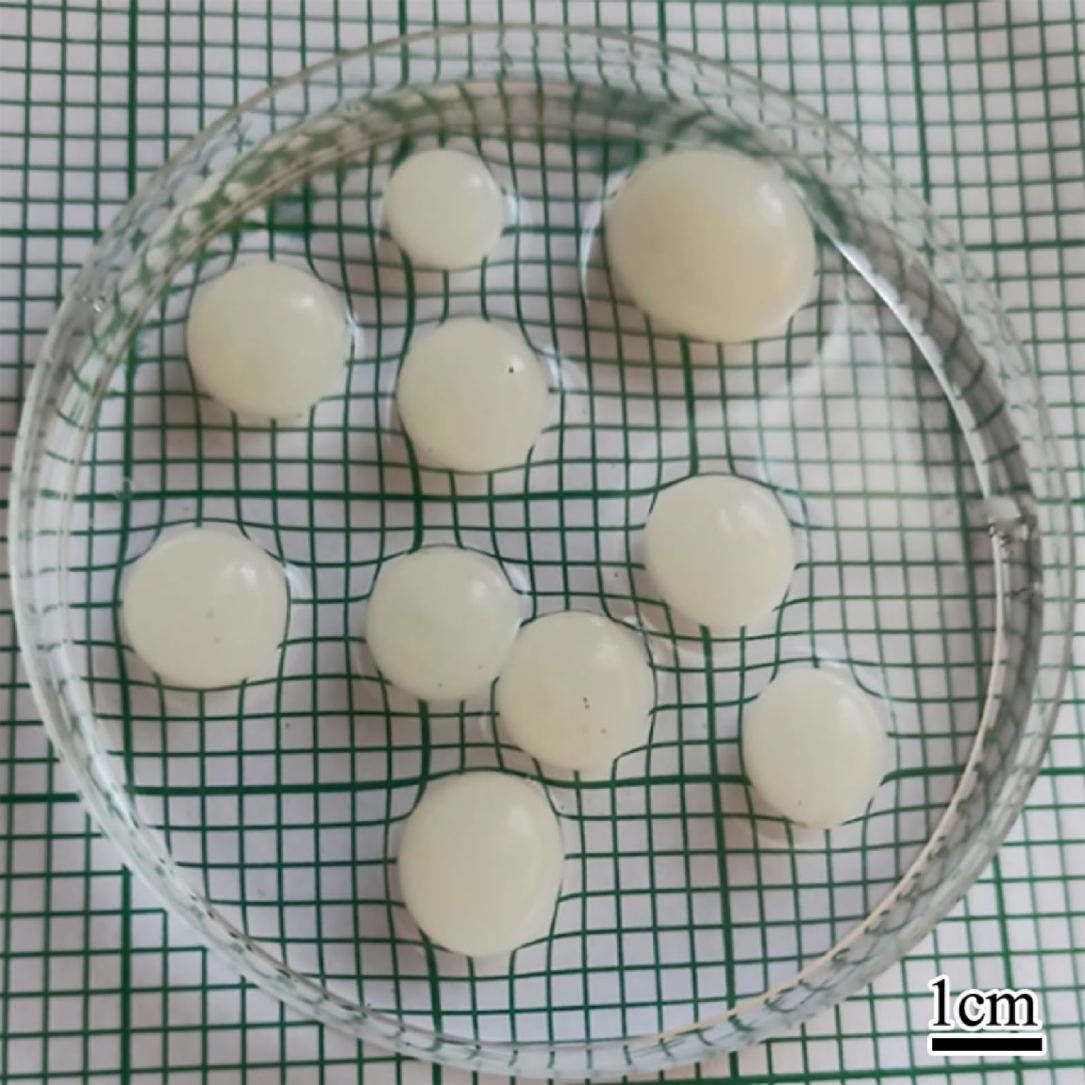
*A. niger* large pellets with diameters 5 to 28 mm formed at 26 ˚C, 10 g/L glucose, 1 × 10^6^ spores/L, and pH = 4 with shaking at 110 rpm (scale bar 1 cm)

The experimental results confirmed the accuracy of the model, depending on the biovolume obtained was then compared with the predicted values suggested by the model, as shown in Table 6, resulting in large pellets with diameters of 5 to 28 mm by culturing 1 × 10^6^ spore suspension conc. At 26 °C, 10 g/L glucose, and pH = 4 with shaking at 110 rpm speed (Fig. 10).

**Table 6:** The actual and predicted values of pellets’ biovolume

Parameters				Actual value	Predicted value
PH	Temp.	Spore.	Glucose	Pellets biovolume (cm^3^/L)	
4	26	10^6^	10	48.5	46.7

### Fungi and algae co-culture

#### Spore-assisted harvest method

According to Table 7: Matrix and responses of the 2-level full factorial design for harvesting of *C. vulgaris* using *A. niger* fungal spores. It appears that a two-level full factorial design was used to study and optimise factors (glucose concentration, spore suspension concentration, and pH) that affected algal fungal co-culture, resulting in 8 distinct runs. However, no significance was found between the factors.

**Table 7:** Matrix and responses of the 2-level full factorial design for harvesting of *C. vulgaris* using *A. niger* fungal spores

Run	Variables			Respone	
	pH	Spore suspension concentration (spores/L)	Glucose (g/L)	Harvest efficiency %	Pellets count (biovolume)/ L
1	6	5	2	80	ND
2	6	9	10	100	ND
3	4	5	10	38.46	ND
4	6	5	10	76.9	ND
5	4	9	10	69.2	60 (29.4 cm^3^/L)
6	6	9	2	99.23	ND
7	4	5	2	74.69	ND
8	4	9	2	44	160 (46.8 cm^3^/L)

*ND: Not detected pellet biovolume means no intact pellets to be measured or harvested, while at the same time, it still can precipitate the algal cells on the flakes’ wall

In runs 2 and 6, respectively, a high harvest efficiency of 100% and 99.23% was observed, but this occurred as a precipitation of the fungal-algal co-culture or loose structure, with no pellets formed. This makes it difficult to remove the microorganisms (fungi and algae) in the form of pellets from the water after using it to treat wastewater.

In runs no. 7 and 8, pellets were formed, but the efficiency of harvest was low, reaching only 74.69% and 69.2% of algal cells, respectively. This suggests that the spore-assisted method to form algal fungal pellets may not be the most effective way to harvest the algal cells.

#### Pellets-assisted harvest method

To improve the efficiency of pellets in harvesting algal cells, *A. niger* pellets were cultivated on *C. vulgaris* culture to study the effect of glucose concentration, initial algal optical density (O. D), and the ratio of pellets biovolume/L of algal culture. A 2-level full factorial design was used to vary these factors at low (−) and high (+) levels, resulting in 8 distinct runs. The target responses to be optimised are displayed in Table 8: Matrix and responses of the 2-level full factorial design for harvesting of *C. vulgaris* using *A. niger* fungal pellets.

**Table 8:** Matrix and responses of the 2-level full factorial design for harvesting of *C. vulgaris* using *A. niger* fungal pellets

Run	Variables			Response
	Algae initial OD	Pellets biovolume (cm^3^/^/^L)	Glucose (g/L)	Harvest efficiency %
1	2	107.17	0	48.91
2	1	107.17	10	94.58
3	1	321.5	0	96.60
4	2	321.5	0	89.18
5	1	321.5	10	**100**
6	1	107.17	0	29.49
7	2	321.5	10	**100**
8	2	107.17	10	93.99

**Fig. 11:**
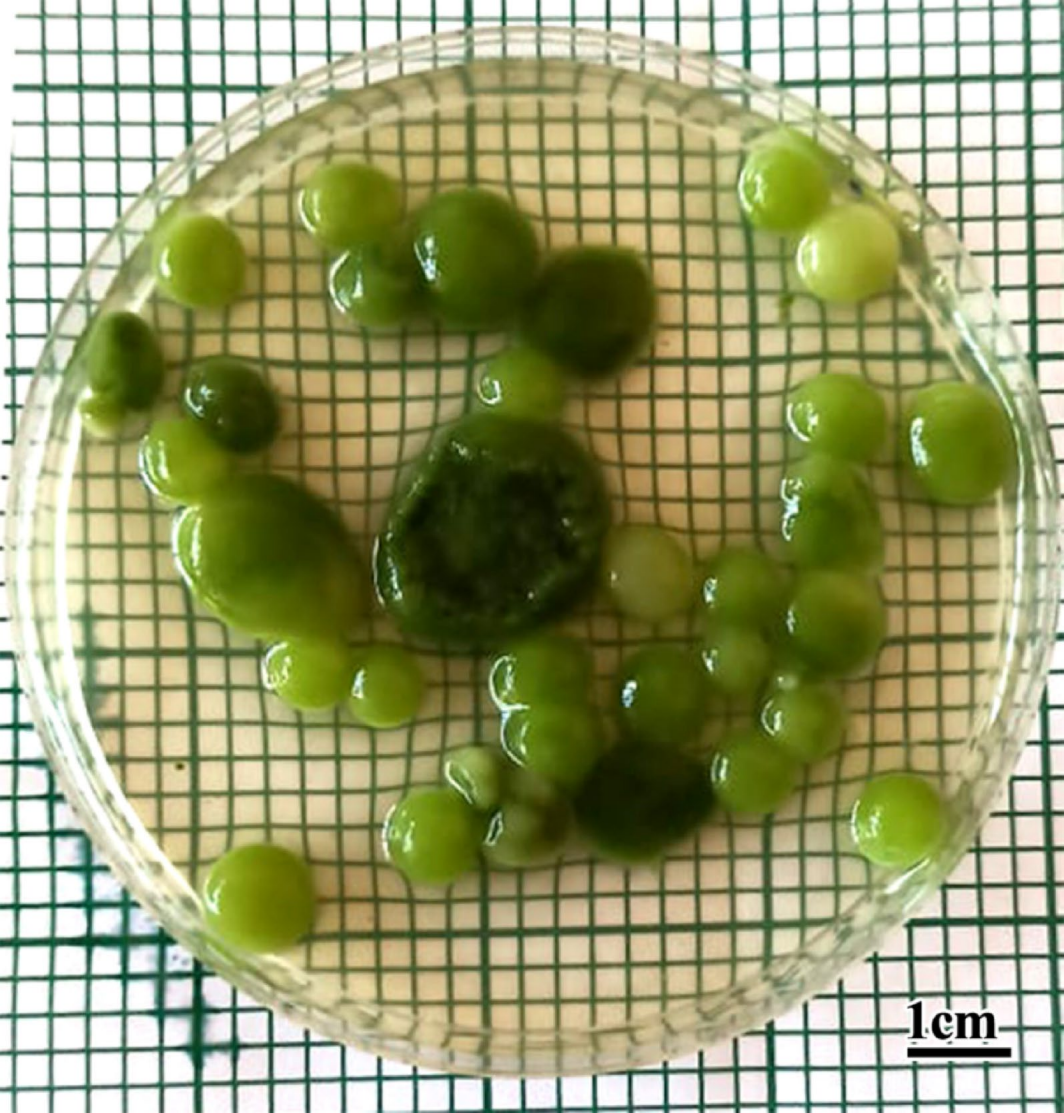
Illustrate the highest efficiency (100%) of *A. niger* preformed pellets to harvest algal isolate *C. vulgaris* (scale bar = 1 cm)

The highest harvest activity was achieved in run No.5 and run No. 7, as harvest efficiency was up to 100% (Fig. 11). However, the lowest harvest efficiency was achieved in run No.6, as harvest efficiency was 29.49%.

The most significant factors affecting harvest efficiency were detected at α = 0.1 (Table 9). Significant factors were marked with an asterisk, showing the most significant factors that affect the efficiency of algal cell harvest using fungal pellets at a 90% level of confidence. The R^2^ value of 99.61% indicated a good fit of the regression model.

**Table 9:** Statistical analysis of 2-level full factorial designs, the regression coefficient, T-value, and *P*-value for each variable on harvest efficiency at a 90% level of confidence at level of confidence

Term	Effect	Coef	T-Value	*p*-Value
Constant		81.59	51.85	0.012
Algal OD	2.85	1.43	0.91	0.531
Glucose	29.70	14.85	9.44	0.067*
Pellets biovolume/L of algal culture	31.10	15.55	9.88	0.064*
Algal OD*Glucose	− 6.56	− 3.28	− 2.08	0.285
Glucose*Pellets biovolume/L of algal culture	− 23.99	− 11.99	− 7.62	0.083*
Algal OD*Glucose*Pellets biovolume/L of algal culture	6.86	3.43	2.18	0.274
Algal OD *Pellets biovolume/L of algal culture	–	–	–	> 0.1

The R2 value of 99.61% indicated a good fit of the regression model, showing the effect and an asterisk for significance

Table 9 showed that adding glucose with a concentration of 10 g/L had a significant positive effect on harvest efficiency, with a *p*-value of 0.067. Additionally, the pellets biovolume used per L of algal culture also had a significant positive impact on harvest efficiency, with a *p*-value of 0.064 Furthermore, there was a significant interaction effect between glucose concentration and the used pellets’ biovolume, with a *p*-value of 0. 083 as it was observed that in absence of glucose when using 107.17 pellets biovolume cm^3^/L the harvest efficiency was 29% and 48.91% at runs 6 and 1, respectively while in presence of glucose at same conditions it reaches 94.58 and 93.99% at runs 2 and 8, respectively. For using 321.5 pellets biovolume/L of algal culture and glucose (10 g/L) in runs 5 and 7, they reached 100% harvest efficiency. In contrast, in the absence of glucose, the harvest efficiency was only 96% and 89.18% at runs 3 and 4, respectively.

**Fig. 12:**
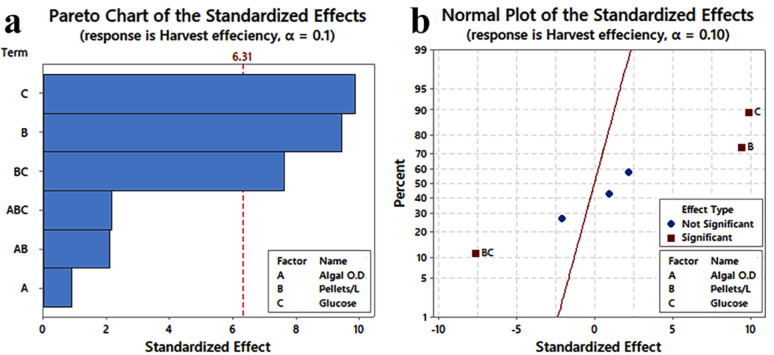
**a** Pareto chart showing the effect of each factor on harvest efficiency, **b** Normal plot of the standardized effect showing the significance of each variable on harvest efficiency

Additionally, the initial algal OD exhibits unclear effects or trends, and consequently, no significance is observed based on the analysis using the Pareto Chart. The variables are arranged in descending order according to their effects, with the variables that cross the red line being statistically significant (Fig. 12a). The normal plot of the standardised effect shows the significant impact of each variable on harvest efficiency. The presence of the variable on the left side of the line represents the statistically negative effect of the variable. In contrast, the presence of the variable on the right side represents the statistically positive effect of the variable. Glucose and the used surface area have a significant positive impact on harvest efficiency (Fig. 12b).

### Wastewater treatment

#### Characteristics of wastewater

Chemical and physical properties are shown in Table 10.

**Table 10:** Wastewater chemical and physical properties like PH, TDS, phosphate, ammonium concentration, and also different elements concentration like Fe, Co, and Ca, etc., which are measured using ICP

Parameter	Concentration average ppm
pH	7.5 ± 0.5
TDS	480 ± 10 ppm
Condactivity	682 ± 15 ppm
Salinity	326 ± 4 ppm
PO_4_^2−^	10 ± 3 ppm
NH_4_^2−^	37 ± 17 ppm
Al	13.653 ppm
V	N.D
Hg	0.731 ppm
Ag	1.111 ppm
B	5.596 ppm
Ba	5.224 ppm
Ca	186.607 ppm
Cd	0.109 ppm
Co	0.278 ppm
Cr	2.892 ppm
Cu	5.569 ppm
Fe	21.523 ppm
Ga	N.D
In	N.D
Li	N.D
Mg	28.963 ppm
Mn	0.713 ppm
Ni	N.D
Pb	0.883 ppm
K	72.655 ppm
Sr	0.698 ppm
Zn	5.683 ppm
As	N.D
Na	73.013 ppm
Bi	1.074 ppm
Se	23.956 ppm

N.D. means not detected (below detection limit)

#### Wastewater as a medium for algae growth

**Fig. 13:**
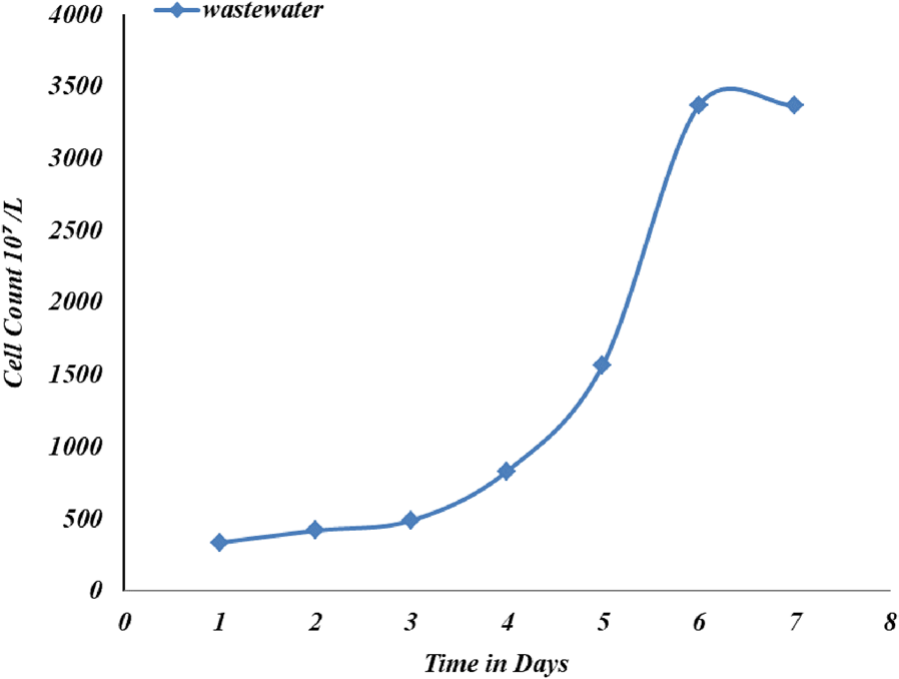
Growth curve of C. *vulgaris* on wastewater

*C. vulgaris* showed the ability to grow on wastewater as a medium and reach its maximum growth in 6 days, and the maximum cell count was 3384 × 10^7^ cells/L, as shown in Fig. 13.

#### Nutrient removal efficiency

Nutrient removal in the form of ammonia and phosphate uptake was analyzed after the growth of *C. vulgaris* on wastewater and harvested with *A. niger* pellets. Glucose and pellet biovolume/L of algal culture, which have a positive effect on harvest efficiency, were studied for their impact on wastewater treatment ability.

**Fig. 14:**
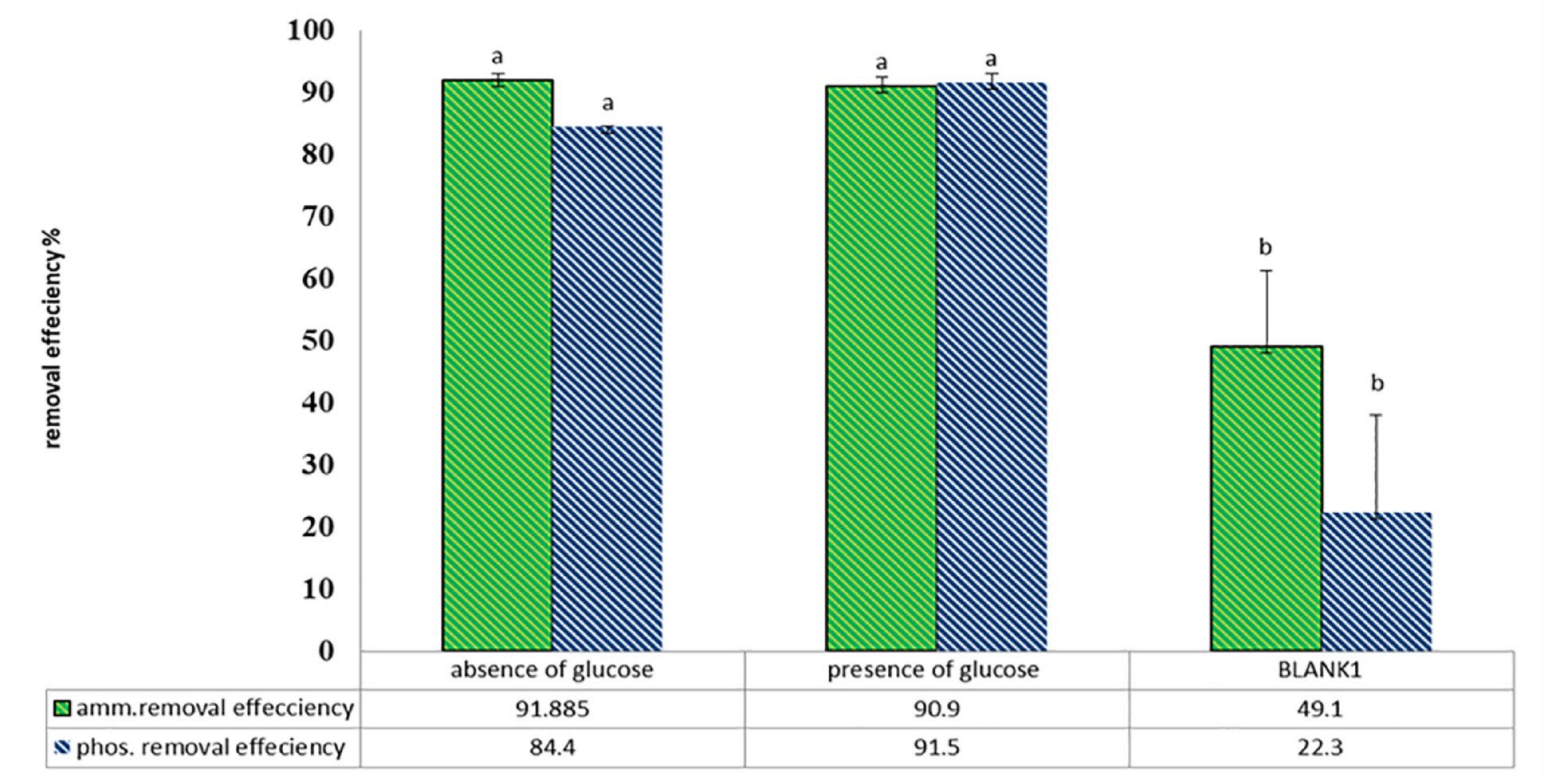
Glucose effect on removal efficiency after wastewater treatment using *C. vulgaris*, then harvested using *A. niger* pellets

For the glucose effect, Table 11 revealed that this method demonstrated remarkable effectiveness in removing ammonium and phosphate, irrespective of the presence or absence of glucose. Specifically, the removal efficiency for ammonium was 91.88% in the absence of glucose and 90.9% in its presence, while the blank showed only 49.1%, whereas the phosphate removal efficiency was 84.4% without glucose, 91.5% with glucose, and only 22.3% in the blank group (Fig. 14).

**Table 11:** Concentrations (ppm) of nutrients in wastewaters and removal efficiency after treatment using *C. vulgaris*, then harvested using *A. niger* pellets in the presence and absence of glucose for 24 h

Treatment	At zero time	Blank after treatment	Absence of glucose	Presence of glucose
Ammonia concentration (ppm)	45.78	23.26	3.7	4.16
Ammonia removal efficiency(%)	–	49.10%	**91.80%**	90.91%
Phosphate concentration (ppm)	10.89	8.45	2.58	0.92
phosphate removal efficiency(%)	–	22.30%	84.40%	**91.51%**

**Fig. 15:**
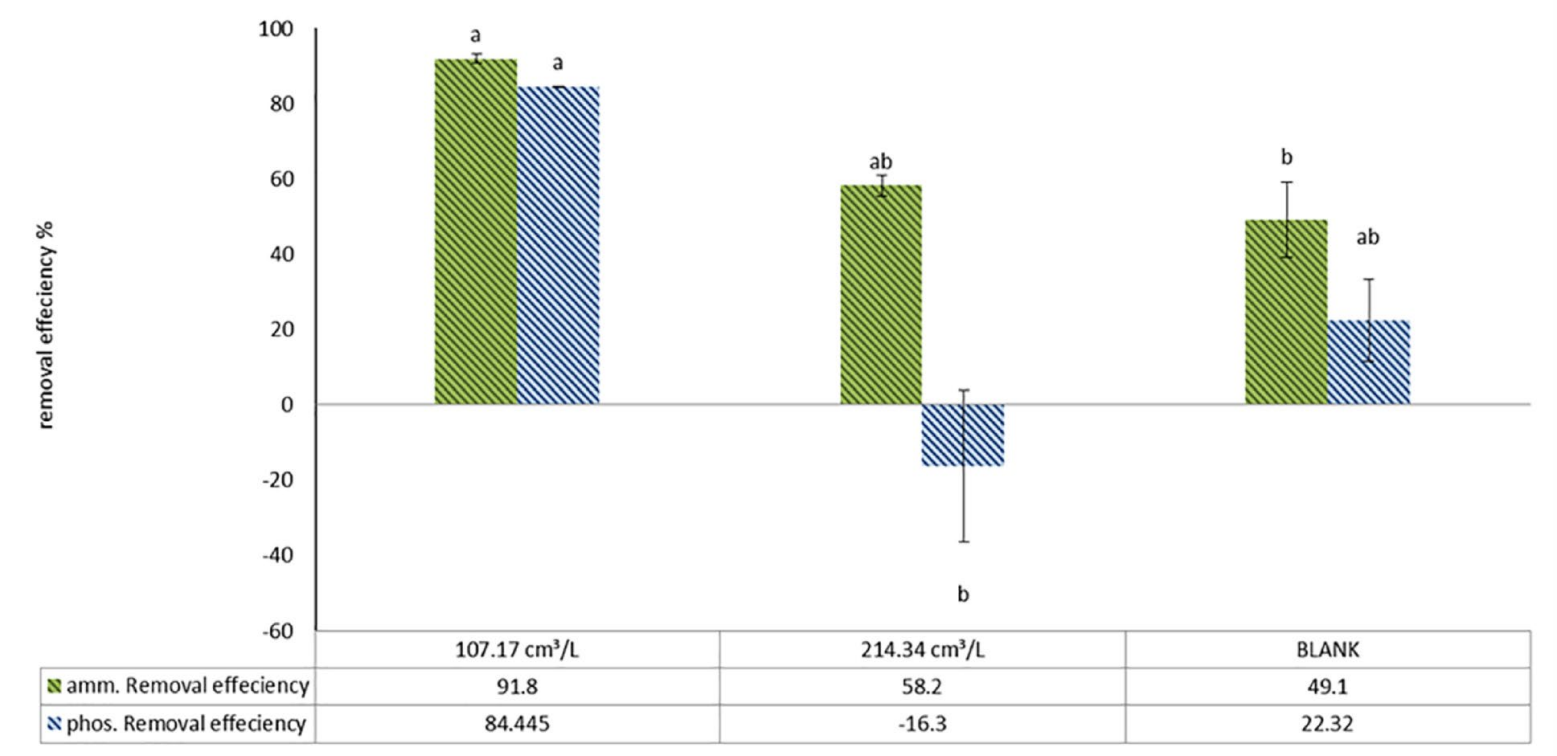
Pellets biovolume cm^3^ /L effect on removal efficiency after wastewater treatment using *C. vulgaris*, then harvested using *A. niger* pellets

For the pellets biovolume/L of algal culture effect, an inverse relationship between high pellet biovolume and treatment efficiency was detected as it is found that using 321.5 cm³/L resulted in cloudy water interrupt measurement of nutrients like phosphate and ammonium so it was excluded, on the other hand Table 12 reveals that ammonium removal rates are significantly higher at a biovolume of 107.17 cm³/L (91.8%) compared to 214.34 cm³/L (58.2%), with the control (blank) group showing only 49.1%. For phosphate, the removal efficiency was 84.4% at 107.17 cm³/L, which is the maximum value, while increasing the biovolume to 214.34 cm³/L resulted in a negative effect of − 16.3% and the control group achieved just 22.3% (Fig. 15).

**Table 12:** Concentrations (ppm) of nutrients in wastewaters and removal efficiency after treatment using *C. vulgaris*, then harvested using *A. niger* using fungal pellets biovolume per liter of algal culture for 24 h

Treatment	At zero time	Blank after treatment	107.17 cm³/L	214.34 cm³/L
Ammonia concentration (ppm)	45.78	23.26	3.71	19.12
Ammonia removal efficiency(%)	–	49.10%	*91.87*%	58.23%
Phosphate concentration.(ppm)	10.89	8.48	1.69	12.67
phosphate removal efficiency(%)	–	22.32%	**84.45%**	− 16.37%

Second stage (using fungal-algal preformed pellets) and its effect on wastewater treatment.

**Fig. 16:**
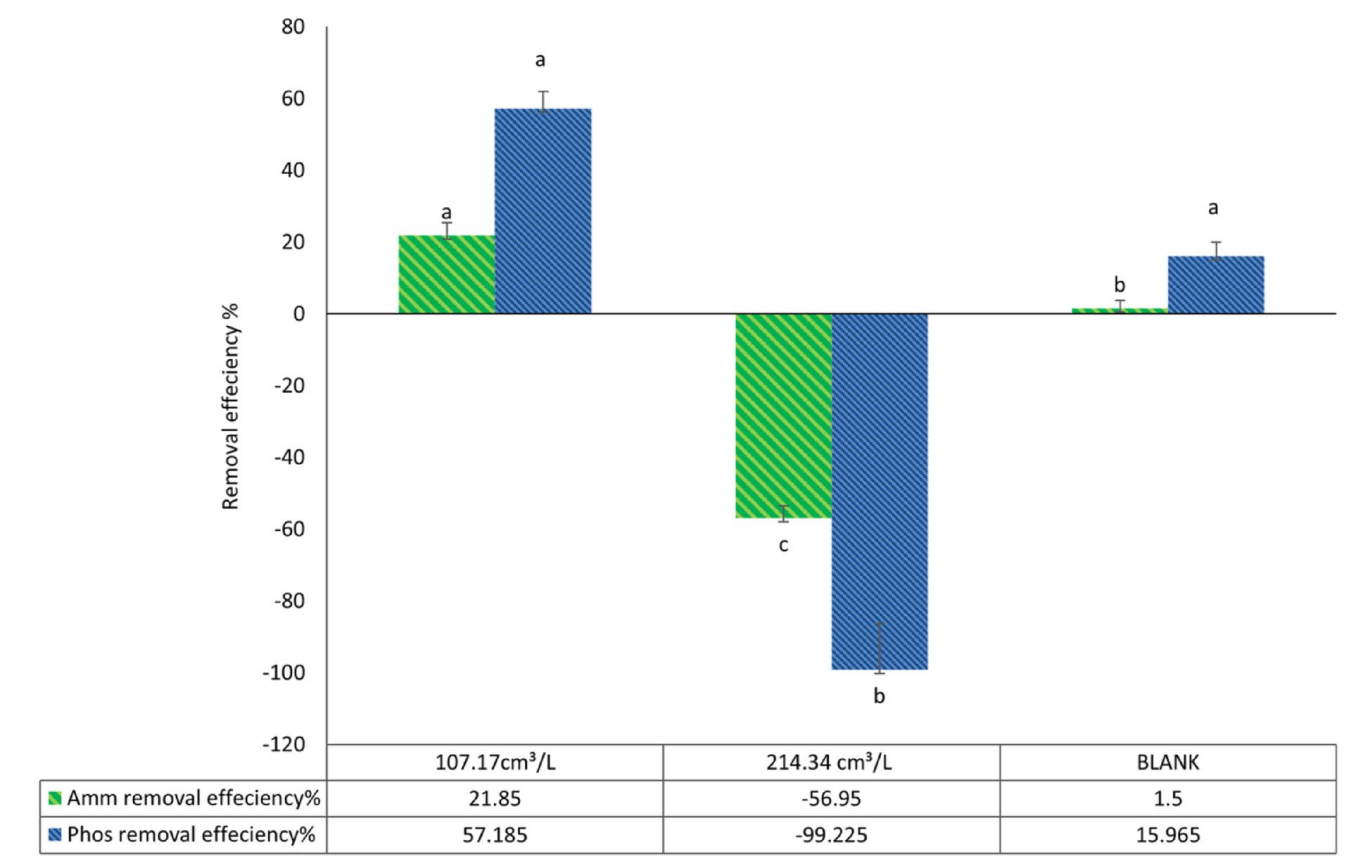
Second stage (using fungal-algal preformed pellets) and its effect on wastewater treatment

In this stage, the resulting fungal-algal pellets from the first stage were reused to treat wastewater. It is found, as shown in Table 13, that by using 107.17 cm³/L of pellets, it can remove up to 21.85% of ammonium and 57.18% of phosphate after 24 h; in contrast, it reaches only 1.5% of ammonium removal and 15.9% phosphate removal in the blank sample (Fig. 16). There is an undesirable effect for increasing the biovolume when it was increased to 214.34 cm³/L. There is not only a negative effect on the treatment process, but it is also observed that using 321.5 m³/L turbidity of water increased, and that some algal cells were dissociated from fungal pellets and restabilized in suspension, so phosphate and ammonium readings can’t be measured at this value.

**Table 13:** Concentrations (ppm) of nutrients in wastewater and removal efficiency after treatment using *A.niger*-*C. Vulgaris* performed pellets for 24 h

Treatment	At zero time	Blank after treatment	107.17 cm³/L	214.34 cm³/L
Ammonia(ppm)	21.03	20.73	16.43	33.015
Ammonia removal efficiency	–	1.50%	*21.85%*	− 56.95%
Phosphate (ppm)	6.072	5.1	2.582	12.1
phosphate removal efficiency	–	15.97%	*57.19%*	− 99.22%

### Lipid extraction

**Fig. 17:**
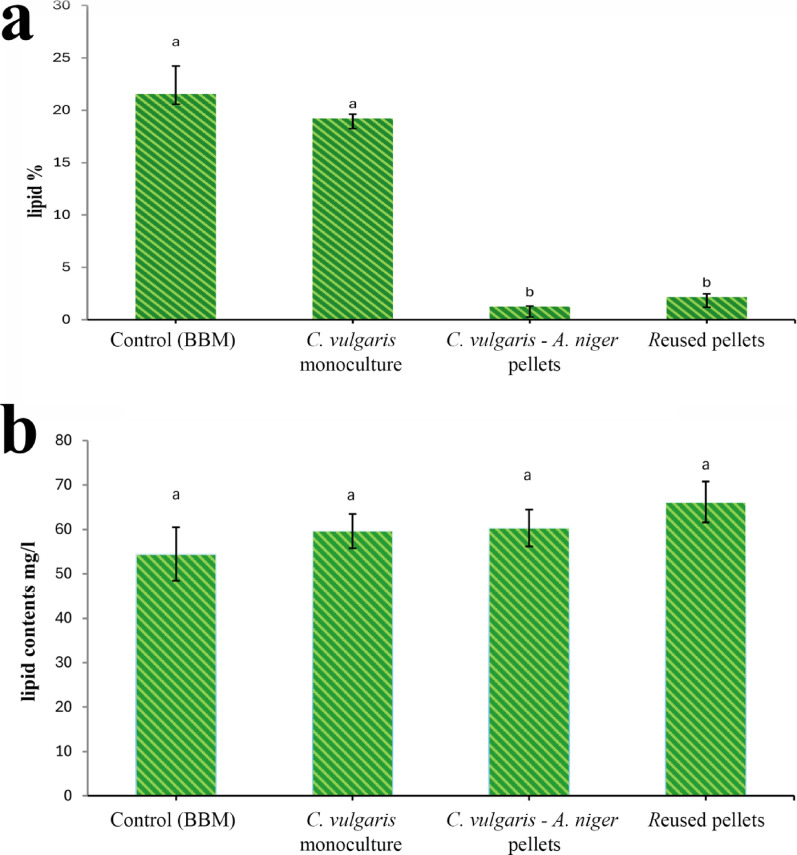
Lipid content (**a**: lipid percentage to dry weight,** b**: lipid in mg/l) of *C.vulgaris*: cultivated in BBM and wastewater and subsequently harvested using *A. niger* pellets

Unialgal culture of *C.vulgaris* on BBM medium (control) showed lipid content of 54.44 ± 10 mg/L with 21.5% of dry weight biomass, while after being treated with wastewater, its yield was 59.6 ± 5 mg/L with a percentage of 18.9% of its dry weight. *C.vulgaris-A.niger* pellets showed high dry weight biomass values with lipid content of 60.3 ± 6 mg/L at stage one, 66.15 ± 6 mg/L at stage two, and with lipid percentage of 1.2 ± 0%, 2.1 ± 0.4% for stages one and two, respectively (Fig. 17a, b).

## Discussion

This study aims to enhance the efficiency of algal cell harvest using fungal-formed pellets as a cost-effective technique for algal harvesting using *A. niger*, which exhibited high pelletisation efficiency when grown on BBM media, which is considered a suitable medium for pellets formation because it contains a high nitrogen source in addition to high Na^+^, Ca^2+^, and Mg^2+^ induce pellet formation process [45]. Furthermore, Mg^2+^ and Fe^2+^ are trace elements that influence the size and number of mycelial pellets [45]. Also, K_2_HPO_4_ was necessary for high harvesting effectiveness [36]. On the other hand, in another study, the presence of KH_2_PO_4_ did not inhibit pellet formation, but it helped to form smooth-appearing pellets [20]. Also, organic carbon sources such as glucose were added to help the fungus grow, and its pelletisation process went well.

### Fungal pelletisation

There are two hypotheses initially proposed to explain pellet formation: the non-coagulation type, where spores germinate and directly grow into pellets, and the coagulation type, where spores quickly gather and germinate into microspheres containing multiple condensed spores. *Aspergillus* species, including *A. niger*, typically follow the coagulation mechanism, with pellets formed from agglomerates of numerous spores [21]. On the other hand, it is suggested that the variety *A. niger* Y3 has these two modes of pellet formation at the same time during growth [45]. The fungal pelletisation involves electrostatic interactions, hydrophobicity, and specific interactions among spore wall components [66], which may explain the ability of *A. niger* mycelium to aggregate instead of disperse in water and form pellets.

#### Factors affecting the formation of fungal pellets

The optimisation process for *A. niger* pellet formation involved studying various factors individually to identify the most effective conditions. The current study’s data analysis revealed that the optimal conditions for pellet formation were pH 4, a temperature of 26 °C, spore suspension concentration of 1 × 10^6^, 1 × 10^7^ spores/L have the same significance, and also glucose concentration of 10 g/L. In contrast, regarding the individual factors studied, it was found that pH 4 was favorable for pellet formation. This result lines up with a similar study that also confirmed the formation at pH 4 [7], which shows that pellet formation was due to the acidic conditions that promote fungal hyphae growth and provide more binding sites [67], speeding up the fungal spore aggregation [66].

Through fungal growth, the culture pH dropped significantly and reached 3.36 due to the excretion of acids in mycelium metabolism [53]. However, extreme acid conditions should be avoided to inhibit protein inactivation and algal cell lysis, and at the same time, they are not favorable for fungal pellet formation [68–70].

Moreover, temperature directly affects the pellets’ formation and morphology [35]. In the current study, temperatures of 26 and 30 °C have the same significant effect as 35 °C in the formation of more stable, smooth, and large pellets. In agreement [52], showed no significant differences in *Rhizopus oryzae* pellet biomass yield when temperatures were between 22 and 33 °C, while the biomass yield at 38 °C was significantly lower. This may be because of the high requirement of cell maintenance under high-temperature conditions [52]. The initial spore suspension concentration was also found to influence fungal pellet formation. It varies depending on the strain, as cells’ quantity and shape directly affect pellet formation, morphology, and growth in submerged cultures [35]. The production of fungal pellets is usually completed with a spore suspension concentration of less than 1 × 10^11^ spores/L [71]. In another study by Chen et al. [72] on *Penicillium* sp., the pellets were formed when the initial spore suspension conc was 1 × 10^6^ spores/L, which was a similar concentration to the current study (1 × 10^6^ and 1 × 10^7^ spores/L), showing the best results for pellet formation. In contrast, for glucose concentration, 10 g/L glucose showed optimum significant concentration because it is utilised as an organic carbon source to provide carbon atoms for many metabolic products that support fungal growth, in agreement with other previous studies that mentioned *A. lentulus* FJ172995, *A. terreus*, * Polyporus* sp. [73], and *Pencillium* sp. [34] as good pelletisation fungi when 10 g/L glucose was used.

#### Optimisation of parameters on A. niger pellet formation

According to the Box-Behnken experimental (*p*-value ≤ 0.05), which means the model can simulate the actual situation very well. In contrast, the model demonstrates statistical significance *p* = 0.02, which suggests that the chosen factors are impactful, marginal lack of fit *p* = 0.0461 suggests additional variables intervals or interactions may need to be broaden. For example, the optimum pH may occur at pH < 4 with a slight difference from an individual experiment, but it will be a highly acidic medium and is not feasible for large-scale application and algal growth, as low pH values negatively affect algal growth and the photosynthesis process in the cells [69, 70].

### Fungi and algae co-culture

The effectiveness of two microalgae harvesting methods, fungal spore-assisted and fungal pellet-assisted, was investigated. When testing *A. niger* spores directly with *C. vulgaris.* in agreement with Zhou W, Min M, Hu B, Ma X, Liu Y, Wang Q, Shi J, Chen P and Ruan R [33] who concluded that initial pH is an essential factor for the co-culturing of *Aspergillus* sp., in the current study, the use of a factorial design for optimisation did not result in a significant improvement in the harvest efficiency of the algal-fungal co-culture and pellet formation at the same time. At pH = 6, no pellets were observed. However, at pH = 4, some pellets were observed with a maximum harvest efficiency of 69.2%, or with a maximum harvest efficiency of 74.69% as precipitated loose structures that were difficult to collect and remove from wastewater, as they never remained intact and disrupted easily during the harvesting process.

The fungal pellet-assisted method showed an excellent result in terms of harvesting efficiency of *A. niger* and *C. vulgaris*, achieving up to 100% within 18 h, which is considered both time and cost savings. This method is more effective than spore-assisted methods, as observed in other studies [34].

The formation of fungal-algal pellets can occur through the interaction between fungal spores and microalgae cells at any stage of the pellet formation process [39]. The process of fungal pellet formation involves electrostatic interactions, as charge neutralisation is considered the primary mechanism, where positively charged fungi neutralise the negative surface charge of microalgae cells [74], leading to their adsorption protein interactions. Also, Bhattacharya et al. [75] concluded, according to FTIR analysis of fungal-algal pellets, that the C-N groups obtained from amino sugars like N-galactosaminogalactan and acetylglucosamine, and C–H groups play a critical function in the fungal interaction with microalgae, and the exopolysaccharide bond also plays a role in this process. Another study explained that the attachment of microalgae to fungal hyphae can be due to sticky exopolysaccharides secreted by the fungi [76]. Divalent calcium and magnesium ions have also been found to affect flocculation while altering the zeta potential of cells, facilitating their attraction and resulting in efficient bio-flocculation [42]. Furthermore, surface protein-mediated, calcium-bridge-dependent, and hydrophobic interactions have been identified as part of the flocculation mechanism in microalgae-fungal pellets [77].

The fungal pellet-assisted method has several advantages, including a shorter harvesting time (18 h) without the need for co-culture with algae spores and reduced physiological effects of fungi on microalgae [34]. This promotes it as an economically and environmentally friendly bioflocculation method, leading to microalgae harvest efficiencies of 100% and 94% with glucose supplementation, where fungal-algae pellets can be combined with simple filtration for microalgae harvest.

Notably, previous studies have also utilised glucose as a carbon source during the pelletisation process [24, 33, 34, 73, 78, 79], even at spore or pellet-assisted methods, highlighting the importance of organic carbon supply for optimal fungal growth and harvest efficiency during the flocculation period, which can speed up the metabolism of fungal pellet to aggregate microalgae, in agreement with [80] which used sucrose to support microalgal harvesting, and this agrees with the optimisation of the fungal pellet-assisted method, which reported that glucose is a significant factor.

### Wastewater treatment

It is found that microalgae and fungal cells are efficient bio-remediating agents for wastewater treatment [12, 81, 82]. Wastewater can be used as a medium for algal growth, as it contains ammonium, phosphate, iron, copper, and other elements that are essential for algal growth. Also, physical and chemical properties indicate that wastewater can be a suitable medium for algal growth, as shown in Table 10.

The pellet-assisted method demonstrated impressive treatment outcomes, regardless of glucose presence, although glucose is vital in improving harvest efficiency. For the second studied factor, pellets biovolume/L of algal culture, ammonium removal reached 91.8%, and phosphate removal reached 84.4% using *A. niger* pellets at a biovolume of 107.17 cm³/L. In contrast, a higher pellets biovolume of 214.34 cm³/L resulted in lower removal efficiencies of 58.2% for ammonium and − 16.3% for phosphate, while using 321.5 cm³/L resulted in fungal pellets beginning to autolyse and affecting the Stability of pellets and caused cloudy water to appear or significantly increase turbidity, which confuses the ability of phosphate and ammonium measurement, which agreed with Chu R, Li S, Zhu L, Yin Z, Hu D, Liu C and Mo F [12] who declared that unsuitable ratios usually result in unilateral growth of algae or fungi. It might even cause confrontational impacts on removal efficiency, mirroring our turbidity/measurement issues. Besides that, high agitation causes hydrodynamic stress and affects pellet growth regions. In another study, it was mentioned that the presence of dispersed hyphae increased the viscosity of the broth medium [71]. As well, this may have occurred due to nutrient limitations, which may arise due to high pellet volumes; the pellets may aggregate or accumulate densely, reducing contact time between contaminant nutrients and the pellets, which may cause autolysis and reduce microbial metabolites [83], which can affect treatment and explain our observed autolysis. Large biomass can consume oxygen rapidly, and this promotes pellet autolysis and may cause mechanical damage to the pellet [84]. It can attract more microbial contamination, which accelerates pellet breakdown. This suggests an inverse relationship between high pellet biovolume and treatment efficiency, contrasted with harvest efficiency, which tends to increase with higher pellet biovolume in the absence of glucose. However, it can be overcome by the addition of glucose; as with 10 g/L of glucose and a biovolume of 107.17 cm³/L, a harvest efficiency of 94.58% was achieved, as indicated by the interface between two factors, as in run 2, Table 8. In comparison, the control group, which contained only normal flora and received no treatment under the same conditions, showed ammonium and phosphate removals of 49.1% and 22.3%, respectively.

In the second stage of the treatment process, which occurred within just 24 h, the fungal-algal pellets demonstrated notable effectiveness in reducing ammonium and phosphate levels, achieving reductions of 21.85% and 57.18%, respectively, with a biovolume of 107.17 cm³/L. In contrast, the blank group only reduced by 1.5% and 15.9%. However, increasing the quantity of pellets had a detrimental effect; at 214.34 cm³/L, higher pollutant levels were observed, leading to increased ammonium and phosphate readings. Furthermore, at 321.5 cm³/L, the treatment caused turbidity and murky water, indicating that excessive fungal-algal pellets can reduce treatment effectiveness and dissociate some algal cells, which agrees with another study [12]. This trend mirrors the negative impacts noted in the first stage when pellet concentrations were also increased, and a beneficial effect when an adequate biovolume was utilized.

### Lipid extraction

Unialgal culture of *C.vulgaris* on BBM medium (control) showed lipid content of 54.44 ± 10 mg/L with 21.5% of dry weight biomass, while after treatment with wastewater, its yield was 59.6 ± 5 mg/L with a percentage of 18.9% of its dry weight. *C.vulgaris-A.niger* pellets showed high dry weight biomass values with lipid content of 60.3 ± 6 mg/L at stage one, 66.15 ± 6 mg/L at stage two, and with lipid percentage of 1.2 ± 0%, 2.1 ± 0.4% for stage one and two, respectively.

Growing *C. vulgaris* on wastewater causes an induction of lipid content percentage up to 9.5%. In the co-culture experiment for the first stage, there was an induction in lipid content of up to 10.8%. Interestingly, the second stage was 21.5%, which suggests that the microalgae are the primary donors to the total lipid content [82]. Although lipid content slightly increased, lipid percentage to dry-weight cells showed a significant decrease, and this may be due to the high dry weight of pellets.

In conclusion, using *A. niger* pellets as a harvesting method for *C. vulgaris* showed promising results in terms of efficiency and cost reduction compared to traditional harvesting methods. While simultaneously enhancing wastewater treatment in nutrient-rich environments through ammonium and phosphate removal, particularly at a specific biovolume, compared to untreated control groups with normal flora, which demonstrate much lower removal rates. However, excessively increasing the pellet concentration led to reduced treatment efficiencies and turbidity issues, complicating measurements. In contrast, the effect of higher pellet biovolume can enhance harvest efficiency without glucose, while adding glucose significantly improves overall effectiveness. This highlighted the importance of using an adequate biovolume to achieve optimal treatment outcomes in wastewater remediation. Future work should optimize turbidity control and scaling-up using the optimum conditions found in the laboratory study.

## Supplementary Information

Below is the link to the electronic supplementary material.

Supplementary Material 1

## Data Availability

No datasets were generated or analysed during the current study.
