# Supplementary figures and images for: Optimising Chlorella vulgaris bioflocculation by Aspergillus Niger pellets and their application in wastewater treatment and lipid production
Source: Microb Cell Fact. 2025 Nov 7;24:229. doi: 10.1186/s12934-025-02849-z (PMC12593925; doi:10.1186/s12934-025-02849-z)

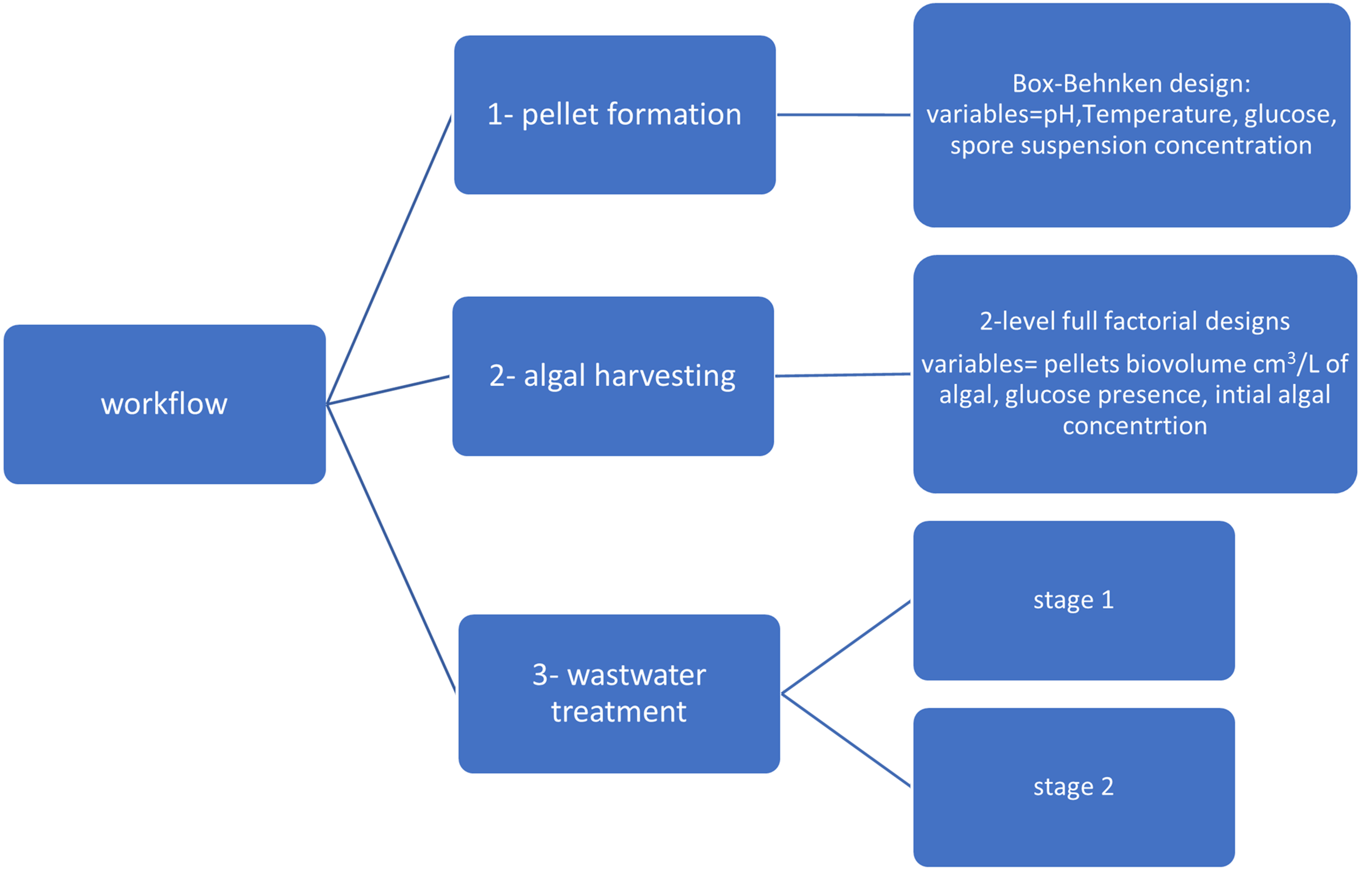

Supplement: Supplementary file 1 — Supplementary Material 1 [file 12934_2025_2849_MOESM1_ESM.png]
